# Genetic origin, admixture, and asymmetry in maternal and paternal human lineages in Cuba

**DOI:** 10.1186/1471-2148-8-213

**Published:** 2008-07-21

**Authors:** Isabel Mendizabal, Karla Sandoval, Gemma Berniell-Lee, Francesc Calafell, Antonio Salas, Antonio Martínez-Fuentes, David Comas

**Affiliations:** 1 Unitat de Biologia Evolutiva, Departament de Ciències Experimentals i de la Salut, Universitat Pompeu Fabra, Barcelona, Spain; 2 Unidade de Xenética, Instituto de Medicina Legal, Facultad de Medicina, Universidad de Santiago de Compostela, and Grupo de Medicina Xenómica, Hospital Clínico Universitario, Santiago de Compostela, Galicia, Spain; 3 Departamento de Biología Animal y Humana. Facultad de Biología, Universidad de la Habana, Ciudad de la Habana, Cuba; 4 CIBER Epidemiología y Salud Pública (CIBERESP), Spain

## Abstract

**Background:**

Before the arrival of Europeans to Cuba, the island was inhabited by two Native American groups, the Tainos and the Ciboneys. Most of the present archaeological, linguistic and ancient DNA evidence indicates a South American origin for these populations. In colonial times, Cuban Native American people were replaced by European settlers and slaves from Africa. It is still unknown however, to what extent their genetic pool intermingled with and was 'diluted' by the arrival of newcomers. In order to investigate the demographic processes that gave rise to the current Cuban population, we analyzed the hypervariable region I (HVS-I) and five single nucleotide polymorphisms (SNPs) in the mitochondrial DNA (mtDNA) coding region in 245 individuals, and 40 Y-chromosome SNPs in 132 male individuals.

**Results:**

The Native American contribution to present-day Cubans accounted for 33% of the maternal lineages, whereas Africa and Eurasia contributed 45% and 22% of the lineages, respectively. This Native American substrate in Cuba cannot be traced back to a single origin within the American continent, as previously suggested by ancient DNA analyses. Strikingly, no Native American lineages were found for the Y-chromosome, for which the Eurasian and African contributions were around 80% and 20%, respectively.

**Conclusion:**

While the ancestral Native American substrate is still appreciable in the maternal lineages, the extensive process of population admixture in Cuba has left no trace of the paternal Native American lineages, mirroring the strong sexual bias in the admixture processes taking place during colonial times.

## Background

At the time of the arrival of Columbus to Cuba in 1492, two different Native American groups inhabited the island: the Ciboneys, spread across the whole island, and the Tainos, mainly occupying the Central and Eastern regions of Cuba [1]. Although not much is known of Ciboney culture including their language, it is known that their economy was based on hunter-gathering (mainly fishing and hunting) and lacked pottery, unlike the Tainos, who were sedentary people living in large settlements and whose culture was supported by technically advanced agriculture. The social organization of the Tainos was based on chiefdoms, in which the *caciques *were the social authority. The Tainos spoke Arawakan, a language belonging to both the Equatorial sub-family and the Equatorial-Tucanoan family [1].

Who first colonized the Caribbean islands is still a matter of debate. Geographical, archaeological and linguistic evidence [2-4], as well as ancient DNA data [5,6] suggest that the Caribbean was most likely populated by successive waves of migration originating in the Lower Orinoco Valley in South America, taking advantage of the close geographical proximity of the islands in the Caribbean. Therefore, the first migratory movement would have involved hunter-gatherer groups arriving around 5000 B.C. (probably the ancestors of the Ciboneys), followed by subsequent migrations of agriculturalists [6]. The Taino Indians would have also migrated from the Orinoco Valley around 1000 B.C., either mixing with or pushing the pre-existent populations towards the West.

**Figure 1:**
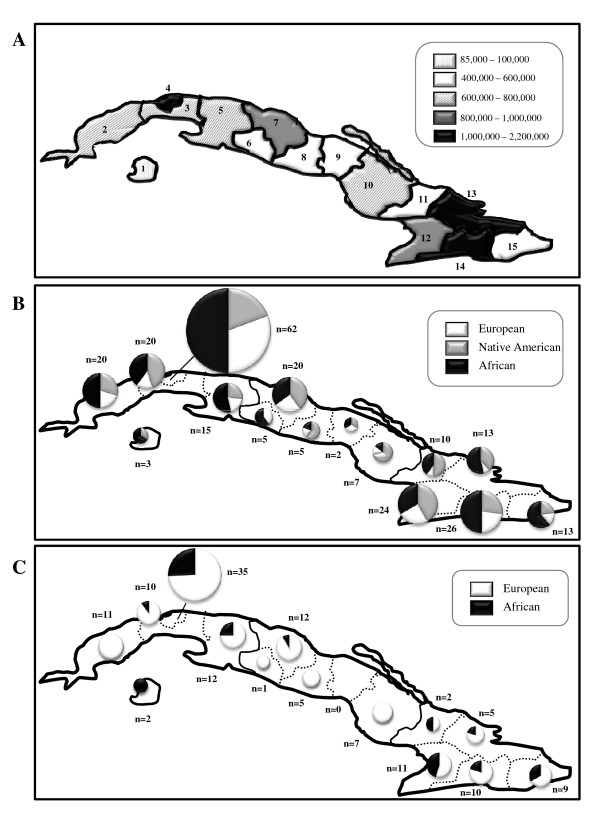
Map of the provinces of Cuba. A) Provinces and their populations according to the 2005 census (Oficina Nacional de Estadística de la República de Cuba): 1 Isla de la Juventud, 2 Pinar del Río, 3 La Habana, 4 Ciudad de la Habana, 5 Matanzas, 6 Cienfuegos, 7 Villa Clara, 8 Sancti Spíritus, 9 Ciego de Ávila, 10 Camagüey, 11 Las Tunas, 12 Granma, 13 Holguín, 14 Santiago de Cuba, 15 Guantánamo. The Departments during the nineteenth century were: West (provinces 1 to 5), Central (provinces 6 to 10) and East (provinces 11 to 15). B) Frequency of mitochondrial lineages: African (black), Native American (grey) and European (white). Circle area is proportional to sample size. C) Y-chromosome lineages found in Cuba: African in black and European in white. Circle area is proportional to sample size (n).

With the arrival of the Europeans both the Ciboney and the Taino populations were drastically reduced within a few generations, a consequence of the harsh slavery conditions, confrontation with settlers, starvation, and infectious diseases. The process of population decrease was more dramatic in the case of the Ciboneys, as this population was already in decline by the time of the Spanish landing in Cuba [7]. Since the sixteenth century, the African slave trade grew steadily over several centuries in Cuba. Slaves were brought mainly as mining laborers to make up for the dramatic demographic decline of Native American people [8]. Overall however, slaves accounted for just a small part of the Cuban population which was mainly European, due to constant migrations from Spain, especially from the Canary Islands. During the second half of the eighteenth century, the introduction of African slaves to Cuba accelerated, dramatically changing the demographic characteristics of the island. In comparison with other American and Caribbean countries, the slave trade to Cuba began earlier and lasted longer. According to revised estimations by Curtin [9], the total number of slaves brought to the island over the whole slave trade period was about 702,000. However, Pérez de la Riva [10] documented more than 1,300,000 slaves since the sixteenth century. The exact origin of only a small fraction of the total African slave population is clearly documented [9], with historical records pointing towards Western (Bight of Benin, North of Congo, Angola, the Bight of Biafra and Sierra Leone) and South-eastern (Mozambique and Madagascar) Africa being the main sources for African slaves. Immigration continued during the nineteenth century when Cuban institutions intensely promoted Spanish immigration, especially from the Canary Islands, reflecting fears of a growing African presence and the desire to "whiten" the Cuban population. In addition, Asian individuals, especially from Bengal and South China, arrived to Cuba in order to substitute the slave labor force when slavery became illegal in the nineteenth century. Around 125,000 Chinese were reported to have arrived to the island to work in conditions of semi-slavery [11]. The arrival of immigrants and slaves to Cuba was not uniform across the island. Thus, during the nineteenth century, the island was organized into three departments (see Figure 1): (a) the Western department was the most populated and had the largest slave population, and subsequently, the largest concentration of laborers due to the development of the sugar industry, (b) the Central part was mainly populated by European livestock farmers, and (c) Eastern Cuba was reported to contain similar amounts of Africans and Europeans [12].

The present-day Cuba presents an attractive enclave in which to study the outcome of a complex history of intricate genetic admixture processes. The high phylogeographic information content of both mitochondrial DNA (mtDNA) and Y-chromosome markers has been investigated at depth in the literature, allowing the reconstruction of past demographic and evolutionary events, such as human migrations and admixture processes. MtDNA and Y-chromosome variation show a strong phylogeographical structure among continental areas, to the point that almost all haplogroups are confined to a single continent and can be used to trace migrations out of that continent. See for instance, figures 9.16 and 9.18 in Jobling et al. [13]. In admixed populations, mtDNA sequences and Y-chromosome haplotypes can be assigned to a continent of origin [14]. In this study we analyzed the complementary information provided by both markers with the aim of surveying (i) the geographic origin of current Cuban ancestors, (ii) the extent of the admixture present among these, and (iii) their differential sexual contribution to the present-day Cuban gene pool.

## Methods

### Samples

Blood stains from 245 unrelated individuals from the general Cuban population were collected blindly with regard to their socioeconomic status in order to avoid ascertainment biases. For each individual, information on the province of origin of the maternal grandmother and paternal grandfather was recorded (see Figure 1) for further analyses on the geographical distribution of the lineages within Cuba. Samples consisted of unrelated healthy blood donors and appropriate informed consent was obtained from all individuals participating in the study. DNA was extracted using standard phenol-chloroform protocols [15].

### MtDNA genotyping

The mtDNA control region was PCR amplified using primers L15996 and H408 [16] following previously published conditions [17]. HVR-I sequences from positions 16024 to 16391 [18](Anderson et al. 1981) were determined for all the individuals (GenBank accession numbers EU649796–EU650040) with the ABI PRISM BigDye Terminator v3.1 Cycle Sequencing kit (Applied Biosystems) according to supplier's recommendations.

Additionally, five SNPs from the mtDNA coding region (10400, 10873, 11719, 12308, and 12705) were genotyped as described in Bosch et al. [19]. These SNPs allowed the classification of mtDNA lineages into six main phylogeographic haplogroups (L, M, N, R, U and HV/H). Two additional sites (7028 and 11251) were also typed in those sequences belonging to HV/H and R as described in Bosch et al. [19]. Information on the control region sequence was used to refine the haplogroup classification.

### Y-chromosome genotyping

Thirty-five Y-chromosome SNPs were typed in the 132 male individuals of the Cuban sample as described by Berniell-Lee et al. [20]. Additionally, the analysis of markers M172, 12f2, M45, M207 and P36 was carried out as described in Bosch et al. [19]. According to these biallelic markers, the samples were finally classified into the main haplogroups and sub-haplogroups according to the Y Chromosome Consortium [21].

### Statistical analysis

Intrapopulation genetic diversity parameters were calculated using the Arlequin package [22]. For mtDNA, the average pairwise differences as well as the weighted intra-lineage mean pairwise difference (WIMP) [23] were calculated. For some analyses, the sample was geographically divided into three groups according to the three departments in which the island was divided during the nineteenth century: West (provinces 1 to 5 in Figure 1), Central (provinces 6 to 10) and East (provinces 11 to 15).

In order to compare the mtDNA sequences obtained, previously published mtDNA data was used (see Additional file 1): 5,370 Africans, 4,147 Native Americans, and 8,645 European lineages. Only variation within the range 16090 to 16365 [18] was used for inter-population comparisons. Each mitochondrial sequence found in Cuba was compared to the corresponding dataset according to the continent of origin of the haplogroup assignation (Africa, America or Eurasia) (see Table 1). Each of the datasets was subdivided into subcontinental regions (see Additional file 1). In order to estimate the putative origin of the Cuban sequences, we calculated the probability of origin of each subcontinental region by a Bayesian approach. The probability of origin of each of the subcontinental region was computed as $p_{0s}=\frac{1}{n}\sum_{i=1}^{n}k_{i}\frac{p_{is}}{p_{ic}}$ where, *n *is the number of Cuban sequences with matching (≥ 1) in the whole continental dataset; k_*i*_, the number of times the sequence *i *is found in the Cuban sample; p_*is*_, the frequency of the sequence *i *in the subcontinental region dataset; and p_*ic*_, the frequency of the sequence *i *in whole continental dataset. In order to provide confidence intervals for each of the estimations for the subcontinental regions, we also computed the standard deviation as $SD(p_{0s})=\sqrt{\frac{p_{0s}\left(1-p_{0s}\right)}{n}}$

**Table 1:** Mitochondrial haplogroup frequencies found in Cuba (245 individuals) grouped according to their phylogeographic origin.

African haplogroups	Cuban individuals	Native American haplogroups	Cuban individuals	West Eurasian haplogroups	Cuban individuals
L0a1	2 (0.8%)	A2	55 (22.4%)	H	22 (9.0%)
L0a1a	2 (0.8%)	B2	5 (2.0%)	I1	1 (0.4%)
L0a2	1 (0.4%)	C1	11 (4.5%)	J*	6 (2.4%)
L1b1	9 (3.7%)	C1d	2 (0.8%)	J2a	1 (0.4%)
L1c	1 (0.4%)	D1	8 (3.3%)	J1b	1 (0.4%)
L1c1	6 (2.4%)			J2	1 (0.4%)
L1c1a	1 (0.4%)			K	2 (0.8%)
L1c2	4 (1.6%)			T*	1 (0.4%)
L2a	19 (7.8%)			T1a	2 (0.8%)
L2a1	2 (0.8%)			T2	2 (0.8%)
L2a1a	3 (1.2%)			T	2 (0.8%)
L2b	1 (0.4%)			U*	2 (0.8%)
L2b1	1 (0.4%)			U4	2 (0.8%)
L2c	3 (1.2%)			U4a2	3 (1.2%)
L2c2	2 (0.8%)			U5a	2 (0.8%)
L3b	11 (4.5%)			V	2 (0.8%)
L3b1	1 (0.4%)			W	1 (0.4%)
L3d	9 (3.7%)				
L3d1	1 (0.4%)				
L3d2	3 (1.2%)				
L3d3	1 (0.4%)				
L3e1	1 (0.4%)				
L3e1a	1 (0.4%)				
L3e2	9 (3.7%)				
L3e2b	2 (0.8%)				
L3e3	2 (0.8%)				
L3e4	1 (0.4%)				
L3f	3 (1.2%)				
L3f1	2 (0.8%)				
L4*	1 (0.4%)				
L4g	1 (0.4%)				
U6a	1 (0.4%)				
U6b1	4 (1.6%)				

TOTAL	111 (45.3%)	TOTAL	81 (33.1%)	TOTAL	53 (21.6%)

## Results

### Phylogeographic analysis of mtDNA lineages in Cuba

A total of 153 different mtDNA sequences were found in the 245 individuals analyzed. Although the mtDNA sequence diversity (0.994 ± 0.001) and the mean pairwise differences (8.102 ± 3.774) in Cubans are relatively high, the WIMP value is low (2.869), suggesting that the sample set is composed of distantly related haplogroups with low to moderate internal diversity. According to the geographical origin attributed to each mtDNA haplogroup, 45% of the mtDNA sequences found in Cubans are of African origin, 33% of Native American origin and 22% of the lineages are of West Eurasian origin (namely, Europe and the Middle East) (Table 1).

Within the African lineages, the vast majority of sequences belong to sub-Saharan L haplogroups (43.3% of the total sample), whereas a small proportion (2% of the total sample) fall into the typical North African U6 haplogroup. Interestingly, most of these U6 sequences (see Additional file 2) belong to the sub-clade U6b1, which is characteristic of Canary Islands [24]. The main U6b1 profile A16163G T16172C A16219G T16311C (three matches in Cuba) is in fact highly prevalent in the Canary Islands (~10%) [24], and outside these islands, it appears only sporadically in some other Latin-American countries such as Uruguay [25].

Haplogroup A2 is the main Native American haplogroup in Cuba (21.9% of the total sample), accounting for 67% of the Native American mtDNA gene pool. It should be stressed that five out of eight sequences belonging to haplogroup D1 and seven sequences out of 13 belonging to haplogroup C have been previously described in extinct Ciboneys [6] and Tainos [5]. Within the small European fraction, the main haplogroup is H, which represents 43% of the European lineages. No East Asian mtDNA lineages [26] were found in the present sample set.

In order to obtain rough estimates for the putative geographic origin of Cuban mtDNA lineages at a more regional continental scale, we searched (identical) matching sequences in different datasets. The Native American lineages found in Cubans were compared to a dataset of 4,147 published Native American sequences (see Additional file 1), divided into three main geographical regions: North (from Alaska to Southern USA; *n *= 2,005), Central (from Mexico to Panama, including the Caribbean islands; *n *= 485) and South America (from Colombia southwards; *n *= 1,657). Only fifteen of the 35 Native American lineages found in Cubans are also found in the American dataset (see Additional file 2). The average of the proportions of Cuban sequences found in each geographical region can be used as a proxy to infer the origin of these lineages within the continent. Thus, the presumed origin of the Cuban Native American sequences could be dissected as follows: 38.7% (SD: 6.9%) to North, 26.7% (SD: 6.2%) to Central and 34.6% (SD: 6.7%) to South America. Although this is a very rough estimate of the putative origin of the lineages within the continent, it shows that the origin of Native American lineages in Cubans cannot be attributed to a single origin within the continent (or that the geographical distribution of mtDNA sequences in America is uninformative).

Using the same approach, the African lineages found in Cubans were compared to a dataset of 5,370 African sequences (see Additional file 1), divided into geographical regions according to [27]: East (*n *= 835), North (*n *= 1,312), South (*n *= 264), South-east (*n *= 416), South-west (*n *= 157), West (*n *= 1,184) and Central (*n *= 1,202). The putative origin of African lineages in Cubans could be mainly traced to Western (30.3% SD: 5.4%), Central (22.2% SD: 4.9%), South-western (18.4% SD: 4.6%) and South-eastern (12.2% SD: 3.9%) Africa, whereas the rest of the continent together (Eastern, Southern and Northern Africa) would presumably account for less than 17%. These figures are in agreement with the proposed origins of African slaves to the Americas [9] and previous findings based on mtDNA analysis. The Cuban African lineages were also compared to African lineages found in the American continent: North (*n *= 1,148), Central (*n *= 83) and South (*n *= 143). The shared sequence pool could also be attributed to Northern (56%) and Central America (26%) rather than South America (18%).

Taking into account a European dataset of 8,645 sequences (see Additional file 1), Cuban profiles were also compared to different geographical regions within Europe: South-west (Portugal, Spain, Italy and the Western Mediterranean islands), West (France and the British Isles), Central Europe (Germany, Switzerland, Austria and the Czech Republic), South-east (Balkan countries) and North (Scandinavia). Following the same rationale used above, the putative origin of the European sequences could be traced mainly to South-western (38.9% SD: 7.6%), Central (20.8% SD: 6.3%) and Western (16.7% SD: 5.8%) Europe. Taking into account that historical documentation testifies to an overwhelming European emigration to Cuba from Spain, this result, although in part corroborating the written legacy, also mirrors the high homogeneity of the European mtDNA variation at the level of haplotype and haplogroup resolution considered here. It is worth highlighting the large amount of sequences shared between Cubans and individuals from the Atlantic islands of Madeira, Azores and the Canary Islands (see Additional file 2).

### Phylogeographic structure of Y-chromosome lineages in Cuba

With respect to the Y-chromosome haplogroups, 78.8% of the chromosomes analyzed can be traced back to the West Eurasian gene pool, whereas the African fraction accounts for 19.7% of Cuban lineages (Table 2); two individuals (1.5%) carried Y-chromosomes of East Asian origin. Among the West Eurasian fraction, the vast majority of individuals belong to West European haplogroup R1 (xR1a). The African lineages found in Cubans have a Western (haplogroups E1, E2, E3a) and Northern (haplogroup E3b2) African origin. Interestingly, we did not observe Native American Y-chromosome lineages, such as those belonging to haplogroups P or Q [28]. Considering that our sample size of Y-chromosomes is 132 individuals (*n*), the highest frequency (*F*) of whatever unobserved haplogroup in the population, with a 95% probability, could be estimated as 1-e^-*Fn *^= 0.95, according to the Poisson distribution. Therefore, it is still possible that Native American haplogroups could be present in the Cuban population with roughly a maximum frequency of 2.3%.

**Table 2:** Y-chromosome haplogroup frequencies found in Cuba (132 individuals) grouped according to their phylogeographic origin.

African haplogroups	Cuban individuals	West Eurasian haplogroups	Cuban individuals	East Asian haplogroups	Cuban individuals
E1	1 (0.8%)	E3b	2 (1.5%)	N/O	1 (0.8%)
E2	2 (1.5%)	E3b1	6 (4.5%)	O2	1 (0.8%)
E3a	13 (9.8%)	G	8 (6.1%)		
E3b2	8 (6.1%)	I	11 (8.3%)		
E3b3	2 (1.5%)	J2	6 (6.1%)		
		K2	2 (1.5%)		
		R1 (xR1a)	67 (50.8%)		
		R1a	2 (1.5%)		

TOTAL	26 (19.7%)	TOTAL	104 (78.8%)	TOTAL	2 (1.5%)

### Population structure

In order to analyze the geographical distribution of the Native American, European and African lineages within Cuba (see Figure 1), each sample was attributed to one of the three regions in which the island was divided: West, Centre and East (see Table 3). No significant differences were found in the proportions of the main mtDNA geographic lineages (χ^2 ^= 7.41, 4 d.f., *P *= 0.116), pointing towards a homogeneous mtDNA genetic landscape across the island. However, significant differences (χ^2 ^= 7.74, 2 d.f., *P *= 0.02) were found among the distribution of the Y-chromosome data among the Western, Central and Eastern regions of Cuba. Indeed, the amount of African lineages in the Central region of Cuba is significantly lower than expected. This finding is in agreement with historical data as European farmers are known to have accounted for the majority of the population in the Central Department.

**Table 3:** Number of individuals found in each Cuban province grouped according to their phylogeographic mtDNA and Y-chromosome origin.

	NATIVE AMERICAN		WEST EURASIAN		AFRICAN		TOTAL	
Provinces	mtDNA	Y-chrom.	mtDNA	Y-chrom.	mtDNA	Y-chrom.	mtDNA	Y-chrom.
Ciudad de la Habana	12 (19.4%)	-	19 (30.6%)	26 (74.3%)	31 (50.0%)	9 (25.7%)	62	35
La Habana	9 (45.0%)	-	3 (15.0%)	9 (90.0%)	8 (40.0%)	1 (10.0%)	20	10
Isla de la Juventud	1 (33.3%)	-	-	2 (100%)	2 (66.7%)	-	3	2
Matanzas	4 (26.7%)	-	3 (20.0%)	9 (75.0%)	8 (53.3%)	3 (25.0%)	15	12
Pinar del Río	6 (30.0%)	-	4 (20.0%)	11 (100%)	10 (50.0%)	-	20	11
TOTAL WEST	32 (26.7%)	0	29 (24.2%)	57 (81.4%)	59 (49.2%)	13 (18.6%)	120	70
								
Ciego de Ávila	1 (50.0%)	-	1 (50.0%)	-	-	-	2	0
Camagüey	5 (71.4%)	-	1 (14.3%)	7 (100%)	1 (14.3%)	-	7	7
Cienfuegos	2 (40.0%)	-	-	1 (100%)	3 (60.0%)	-	5	1
Sancti Spíritus	3 (60.0%)	-	1 (20.0%)	5 (100%)	1 (20.0%)	-	5	5
Villa Clara	8 (40.0%)	-	5 (25.0%)	11 (91.7%)	7 (35.0%)	1 (8.3%)	20	12
TOTAL CENTER	19 (48.7%)	0	8 (20.5%)	24 (96.0%)	12 (30.8%)	1 (4.0%)	39	25
								
Granma	10 (41.7%)	-	6 (25.0%)	6 (54.5%)	8 (33.3%)	5 (45.5%)	24	11
Guantánamo	3 (23.1%)	-	2 (15.4%)	6 (66.7%)	8 (61.5%)	3 (33.3%)	13	9
Holguín	5 (38.5%)	-	1 (7.7%)	4 (80.0%)	7 (53.8%)	1 (20.0%)	13	5
Las Tunas	5 (50.0%)	-	1 (10.0%)	1 (50.0%)	4 (40.0%)	1 (50.0%)	10	2
Santiago de Cuba	7 (26.9%)	-	6 (23.1%)	8 (80.0%)	13 (50.0%)	2 (20.0%)	26	10
TOTAL EAST	30 (34.9%)	0	16 (18.6%)	25 (67.7%)	40 (46.5%)	12 (32.4%)	86	37

## Discussion

In contrast with the popular belief that the ancestral Native American pool in Cuba was totally erased by the massive arrival of Europeans and African slaves and centuries of admixture, and despite the absence of distinct ethnic Native American groups in Cuba, the present results demonstrate the persistence of a substantially high Native American component in the maternal specific gene pool. The presence of an unexpectedly high proportion Native American mtDNA substrate has been described previously in other American populations that also experienced dramatic demographic changes in colonial times, such as Puerto Rico [29,30](Martinez-Cruzado et al. 2001)(Martinez-Cruzado et al. 2005), Brazil [31] and Mexico [32]. The estimated Native American component inferred in Cuba is higher than those estimates based on nuclear markers (<5%) [33]. In addition, the frequency of Native American mtDNA lineages in Cuba is larger than in the English-speaking Caribbean countries (5.4%) [34] as well as in Afro-American populations from Central and South America such as the Garifuna from Honduras (15.9%) and the Chocó people from Colombia (16.3%) [35] (see Additional file 3). In these cases, the indigenous population was even more dramatically replaced by African slaves and to a certain extent by Europeans. Our results differ from a previous independent study carried out in the Cuban province of Pinar del Rio [36], whose authors estimated that 50% of maternal lineages in this province were of European, 46% African, and a maximum 4% of Native American origin. Our results indicate that the Native American mtDNA haplogroup patterns are statistically homogeneous across the island, the maternal Native American substrate being higher than 25% in all provinces. Specifically, in Pinar del Rio we detected 33% Native American maternal lineages, a figure that significantly contrasts (χ^2 ^= 12.32; 2 d.f., *P *= 0.002) with the 4% found in the study by Torroni [36]. This difference highlights the risk of population stratification that can easily show up in case-control disease association studies, for example, leading to an increase of the false positive rate [26,37]. Forensic genetic studies are also sensitive to population stratification. This is especially true in pseudo-ethnic groups such as the 'Hispanics', a term firmly established in American societies, and in particular, in the USA [38]. Although our recruitment scheme was designed to capture a representative sample of the Cuban population, and although blood donations were not rewarded, our sample could include unapparent socioeconomic biases that would distort ancestry estimates.

The origin of Native Americans in the Caribbean, such as Ciboneys and Tainos, is a controversial issue. The present mtDNA Native American haplogroup frequencies in contemporary Cuba differ significantly (*P *< 0.0001) from the haplogroup composition observed in ancient DNA samples from Ciboneys and Tainos. Fifteen ancient specimens of Ciboneys from Cuba have been analyzed [6] and classified into haplogroups C1 (nine individuals), D1 (five individuals), and A2 (one individual), while, in a different study [5], 24 samples of extinct Tainos from the neighboring island of the Dominican Republic were analyzed and classified as C1 (18 individuals) and D1 (6 individuals). Neither haplogroups A2 nor B2 were observed. According to Lalueza-Fox et al. [6], the scarcity of haplogroup A2 and the predominance of lineages C1 and D1 in the Caribbean point towards South America as the origin of both the Tainos and the Ciboneys. However, an argument based only on average continental haplogroup frequencies can be misleading since haplogroup frequencies vary substantially in different present-day Native American populations, either within North, Central, and South America. A process of progressive island colonization coming from the Orinoco Valley and/or from the Yucatan provides an appropriate ground for the action of genetic drift. Intensive episodes of genetic drift are in fact the rule more than the exception in other Native American populations. Over half of the Cuban sequences belonging to haplogroups C1 and D1 described in the present study have been already described in ancient DNA studies [5,6] in a total of 39 individuals (24 Tainos and 15 Ciboneys). However, these sequences are common in Native American populations (from both North and South America) and many represent founding lineages in the continent. In contrast to the hypothesis by Lalueza-Fox et al. (2003), our data suggest that both North and South America could have contributed to the original gene pool of Cuban Native Americans. We anticipate an even more complex scenario where the contribution of other Native American people coming from different continental locations in the post-colonization period could have contributed to the already admixed population. In fact, importation of Native Americans from Central and North America has already been reported [39]. Therefore, sampling effects consisting of merely the existence of close maternal relatedness between the individual analyzed could have contributed to distorting the haplogroup patterns observed in ancient Tainos. This hypothesis would also explain why the predominance of the C1 and D1 haplogroups in these pre-Columbian samples is not observed neither in present-day Cuba nor in Puerto-Rico [29,30], where the Tainos were also the Native inhabitants before the European arrival.

Although the hypothesized Southern origin of the Native American Cuban people as coming from the Orinoco Valley has been historically favored [2-4], our results indicate that a substantial genetic input from Central and North America (e.g. Yucatan or Florida peninsulas) cannot be ruled out. Due to the vulnerability of haplogroup frequencies to genetic drift, the phylogeographic information provided by the sequences is a necessary complementary tool in order to locate the origin of the Cuban sample in the context of the American continent. Thus, the comparison of the Cuban sequences to a dataset of more than 4,000 sequences covering the entire continent suggests a multiregional origin within America since the number of matches was similar for North, Central and South America. We are also aware that phylogeographic information is still of limited use because Native American lineages are scarcely informative for the HVS-I mtDNA control region. A higher molecular resolution based on the analysis of complete genomes (or high throughput mtDNA SNP coding region scans) can be useful to refine phylogeographic inferences [40,41].

Besides the presence of maternal Native American substrate in Cuba, the present results show a strong sexual asymmetry between European males and non-European females in Cuba. In contrast to the 33% Native American presence in the female lineages, no Native American fraction was found in the Y-chromosome haplogroups. This result is in agreement with historical documentation, which records the high prevalence of Native American-'*white mestizos' *in the first generations after the conquest. The European settlers were in vast majority men, and mating between Spanish men and Native American women was not uncommon during the first generations of settlers [7]. Similar sex biases between Native American and European founders have been previously described in Brazil [31,42,43] and Colombia [44]. Regarding the African component, the strong bias between the mtDNA and Y-chromosome haplogroup frequencies is also noticeable. While the African lineages constitute 45% of the total maternal lineages, they are present in only 18% of the Cuban Y-chromosomes. Although extremely high amounts of African slaves were carried to Cuba, the African-born slave population presented extremely high rates of mortality and an unfavorable sex ratio. The '*mulattos' *were considered inferior in the Cuban society since the beginning of the slave trade [7], so that mating between African men and European women was strongly discouraged. In contrast, the mating of European masters and the African slave women was more common.

## Conclusion

This report shows that despite centuries of inter-ethnic mating between people from different continents, the Native American substrate persists in the present-day Cuban population contributing more than a third of the total maternal lineages. We have also described a noticeable European/Native American, as well as European/African sex bias between the paternal and maternal ancestries in Cuba. In addition, the origin of the present day Cuban Native American substrate cannot be uniquely ascribed to South America, having also received an important genetic input from Central and/or North America.

## Authors' contributions

IM carried out genetic laboratory analysis, analyzed the data and drafted the manuscript. KS and GBL helped with the experimental design. FC and AS contributed in data analysis. AMF provided the samples. DC conceived and directed the study. All authors reviewed and commented on the manuscript during its drafting and approved the final version.

## Supplementary Material

Additional file 1 References for the mtDNA sequences included in the datasets used for comparisons to Cuban sequences in the present study. Click here for file

Additional file 2 HVS-I sequences found in Cuba with their haplogroup classification and number of (identical) and matches in the datasets used for comparison Click here for file

Additional file 3 References for the studies on admixed populations used for comparisons. Click here for file

## References

[B1] Dacal-Moure R Rivero de la Calle M Arqueología aborigen de Cuba 1986 La Habana , Editorial Gente Nueva

[B2] Moreira de Lima LJ La sociedad comunitaria de Cuba 1999 La Habana , Félix Varela

[B3] Rouse I Migrations in prehistory 1986 New Haven , Yale University Press

[B4] Rouse I The Tainos 1992 New Haven , Yale University Press

[B5] Lalueza-Fox C Calderon FL Calafell F Morera B Bertranpetit J MtDNA from extinct Tainos and the peopling of the Caribbean Annals of human genetics 2001 65 Pt 2 137 151 10.1046/j.1469-1809.2001.6520137.x 11427174

[B6] Lalueza-Fox C Gilbert MT Martínez-Fuentes AJ Calafell F Bertranpetit J Mitochondrial DNA from pre-Columbian Ciboneys from Cuba and the prehistoric colonization of the Caribbean American journal of physical anthropology 2003 121 2 97 108 10.1002/ajpa.10236 12740952

[B7] Guerra R Manual de historia de Cuba desde el descubrimiento hasta 1868 1971 La Habana , Editorial de Ciencias Sociales

[B8] Kiple KF The Caribbean Slave: A Biological History 1984 Cambridge , Cambridge University Press

[B9] Curtin PD The Atlantic slave trade: a census 1969 Madison The University of Wisconsin Press

[B10] Pérez de la Riva J El monto de la inmigración forzada en el siglo XIX 1979 Ciudad de la Habana , Editorial Ciencias Sociales

[B11] Checa M Hacia una geografía de las primeras migraciones chinas en el Caribe Biblio 3W Revista Bibliográfica de Geografía y Ciencias Sociales Universidad de Barcelona 2007 XII 707

[B12] Le Riverend J Historia Económica de Cuba 1967 La Habana , Instituto Cubano del Libro

[B13] Jobling MA Hurles M Tyler-smith C Human evolutionary genetics: origins, peoples, and disease 2004 Abingdon and New York , Garland Science

[B14] Berniell-Lee G Plaza S Bosch E Calafell F Jourdan E Cesari M Lefranc G Comas D Admixture and sexual bias in the population settlement of La Reunion Island (Indian Ocean) American journal of physical anthropology 2008 136 1 100 107 10.1002/ajpa.20783 18186507

[B15] Sambrook J Fritsch EF Maniatis T Molecular cloning. A laboratory manual. 1989 Second New York , Cold Spring Harbor Laboratory Press

[B16] Vigilant L Pennington R Harpending H Kocher TD Wilson AC Mitochondrial DNA sequences in single hairs from a southern African population Proceedings of the National Academy of Sciences of the United States of America 1989 86 23 9350 9354 10.1073/pnas.86.23.9350 2594772 PMC298493

[B17] Mateu E Comas D Calafell F Perez-Lezaun A Abade A Bertranpetit J A tale of two islands: population history and mitochondrial DNA sequence variation of Bioko and Sao Tome, Gulf of Guinea Annals of human genetics 1997 61 Pt 6 507 518 10.1017/S0003480097006544 9543551

[B18] Anderson S Bankier AT Barrell BG de Bruijn MH Coulson AR Drouin J Eperon IC Nierlich DP Roe BA Sanger F Schreier PH Smith AJ Staden R Young IG Sequence and organization of the human mitochondrial genome Nature 1981 290 5806 457 465 10.1038/290457a0 7219534

[B19] Bosch E Calafell F González-Neira A Flaiz C Mateu E Scheil HG Huckenbeck W Efremovska L Mikerezi I Xirotiris N Grasa C Schmidt H Comas D Paternal and maternal lineages in the Balkans show a homogeneous landscape over linguistic barriers, except for the isolated Aromuns Annals of human genetics 2006 70 Pt 4 459 487 10.1111/j.1469-1809.2005.00251.x 16759179

[B20] Berniell-Lee G Sandoval K Mendizabal I Bosch E Comas D SNPlexing the human Y-chromosome: A single-assay system for major haplogroup screening Electrophoresis 2007 28 18 3201 3206 10.1002/elps.200700078 17703471

[B21] YCC A nomenclature system for the tree of human Y-chromosomal binary haplogroups Genome Res 2002 12 2 339 348 10.1101/gr.217602 11827954 PMC155271

[B22] Excoffier L Laval G Schneider S Arlequin ver. 3.0:An integrated software package for population genetics data analysis Evolutionary Bioinformatics Online 2005 1 47 50 19325852 PMC2658868

[B23] Jansen T Forster P Levine MA Oelke H Hurles M Renfrew C Weber J Olek K Mitochondrial DNA and the origins of the domestic horse Proceedings of the National Academy of Sciences of the United States of America 2002 99 16 10905 10910 10.1073/pnas.152330099 12130666 PMC125071

[B24] Rando JC Cabrera VM Larruga JM Hernández M González AM Pinto F Bandelt HJ Phylogeographic patterns of mtDNA reflecting the colonization of the Canary Islands Annals of human genetics 1999 63 Pt 5 413 428 10.1046/j.1469-1809.1999.6350413.x 10735583

[B25] Pagano S Sans M Pimenoff V Cantera AM Álvarez JC Lorente JA Peco JM Mones P Sajantila A Assessment of HV1 and HV2 mtDNA variation for forensic purposes in an Uruguayan population sample Journal of forensic sciences 2005 50 5 1239 1242 10.1520/JFS2004362 16225241

[B26] Kong QP Bandelt HJ Sun C Yao YG Salas A Achilli A Wang CY Zhong L Zhu CL Wu SF Torroni A Zhang YP Updating the East Asian mtDNA phylogeny: a prerequisite for the identification of pathogenic mutations Human molecular genetics 2006 15 13 2076 2086 10.1093/hmg/ddl130 16714301

[B27] Salas A Richards M De la Fé T Lareu MV Sobrino B Sánchez-Diz P Macaulay V Carracedo A The making of the African mtDNA landscape Am J Hum Genet 2002 71 5 1082 1111 10.1086/344348 12395296 PMC385086

[B28] Bortolini MC Salzano FM Thomas MG Stuart S Nasanen SP Bau CH Hutz MH Layrisse Z Petzl-Erler ML Tsuneto LT Hill K Hurtado AM Castro-de-Guerra D Torres MM Groot H Michalski R Nymadawa P Bedoya G Bradman N Labuda D Ruiz-Linares A Y-chromosome evidence for differing ancient demographic histories in the Americas Am J Hum Genet 2003 73 3 524 539 10.1086/377588 12900798 PMC1180678

[B29] Martínez-Cruzado JC Toro-Labrador G Ho-Fung V Estévez-Montero MA Lobaina-Manzanet A Padovani-Claudio DA Sánchez-Cruz H Ortiz-Bermúdez P Sánchez-Crespo A Mitochondrial DNA analysis reveals substantial Native American ancestry in Puerto Rico Human biology; an international record of research 2001 73 4 491 511 10.1353/hub.2001.0056 11512677

[B30] Martínez-Cruzado JC Toro-Labrador G Viera-Vera J Rivera-Vega MY Startek J Latorre-Esteves M Roman-Colon A Rivera-Torres R Navarro-Millan IY Gómez-Sánchez E Caro-González HY Valencia-Rivera P Reconstructing the population history of Puerto Rico by means of mtDNA phylogeographic analysis American journal of physical anthropology 2005 128 1 131 155 10.1002/ajpa.20108 15693025

[B31] Alves-Silva J da Silva Santos M Guimarães PE Ferreira AC Bandelt HJ Pena SD Prado VF The ancestry of Brazilian mtDNA lineages Am J Hum Genet 2000 67 2 444 461 10.1086/303004 10873790 PMC1287189

[B32] Green LD Derr JN Knight A mtDNA affinities of the peoples of North-Central Mexico Am J Hum Genet 2000 66 3 989 998 10.1086/302801 10712213 PMC1288179

[B33] Alegre R Moscoso J Martínez-Laso J Martin-Villa M Suárez J Moreno A Serrano-Vela JI Vargas-Alarcon G Pacheco R Arnaiz-Villena A HLA genes in Cubans and the detection of Amerindian alleles Mol Immunol 2007 44 9 2426 2435 10.1016/j.molimm.2006.10.017 17123606

[B34] Torres JB Kittles RA Stone AC Mitochondrial and Y Chromosome Diversity in the English-Speaking Caribbean Ann Hum Genet 2007 71 Pt 6 782 790 10.1111/j.1469-1809.2007.00380.x 17596204

[B35] Salas A Richards M Lareu MV Sobrino B Silva S Matamoros M Macaulay V Carracedo A Shipwrecks and founder effects: divergent demographic histories reflected in Caribbean mtDNA American journal of physical anthropology 2005 128 4 855 860 10.1002/ajpa.20117 16047324

[B36] Torroni A Brown MD Lott MT Newman NJ Wallace DC African, Native American, and European mitochondrial DNAs in Cubans from Pinar del Rio Province and implications for the recent epidemic neuropathy in Cuba. Cuba Neuropathy Field Investigation Team Hum Mutat 1995 5 4 310 317 10.1002/humu.1380050407 7627185

[B37] Mosquera-Miguel A Álvarez-Iglesias V Carracedo A Salas A Vega A Carracedo A Milne R de León AC Benitez J Carracedo A Salas A Is mitochondrial DNA variation associated with sporadic breast cancer risk? Cancer research 2008 68 2 623 5; author reply 624 10.1158/0008-5472.CAN-07-2385 18199560

[B38] Salas A Bandelt HJ Macaulay V Richards MB Phylogeographic investigations: The role of trees in forensic genetics Forensic Sci Int 2007 in press 168 1 13 10.1016/j.forsciint.2006.05.037 16814504

[B39] Victoria J La hacienda de Yucatán Historia 2002 16 26 74 83

[B40] Achilli A Perego UA Bravi CM Coble MD Kong QP Woodward SR Salas A Torroni A Bandelt HJ The phylogeny of the four pan-American MtDNA haplogroups: implications for evolutionary and disease studies PLoS ONE 2008 3 3 e1764 10.1371/journal.pone.0001764 18335039 PMC2258150

[B41] Tamm E Kivisild T Reidla M Metspalu M Smith DG Mulligan CJ Bravi CM Rickards O Mártinez-Labarga C Khusnutdinova EK Fedorova SA Golubenko MV Stepanov VA Gubina MA Zhadanov SI Ossipova LP Damba L Voevoda MI Dipierri JE Villems R Malhi RS Beringian standstill and spread of Native American founders PLoS ONE 2007 2 9 e829 10.1371/journal.pone.0000829 17786201 PMC1952074

[B42] Batista dos Santos SE Rodrigues JD Ribeiro-dos-Santos AK Zago MA Differential contribution of indigenous men and women to the formation of an urban population in the Amazon region as revealed by mtDNA and Y-DNA American journal of physical anthropology 1999 109 2 175 180 10.1002/(SICI)1096-8644(199906)109:2<175::AID-AJPA3>3.0.CO;2-# 10378456

[B43] Carvalho-Silva DR Santos FR Rocha J Pena SD The phylogeography of Brazilian Y-chromosome lineages Am J Hum Genet 2001 68 1 281 286 10.1086/316931 11090340 PMC1234928

[B44] Carvajal-Carmona LG Soto ID Pineda N Ortiz-Barrientos D Duque C Ospina-Duque J McCarthy M Montoya P Álvarez VM Bedoya G Ruiz-Linares A Strong Amerind/white sex bias and a possible Sephardic contribution among the founders of a population in northwest Colombia Am J Hum Genet 2000 67 5 1287 1295 10.1086/321216 11032790 PMC1288568

