# Supplementary material for: Genetic origin, admixture, and asymmetry in maternal and paternal human lineages in Cuba
Source: BMC Evol Biol. 2008 Jul 21;8:213. doi: 10.1186/1471-2148-8-213 (PMC2492877; doi:10.1186/1471-2148-8-213)
Supplement: Additional file 1 — References for the mtDNA sequences included in the datasets used for comparisons to Cuban sequences in the present study. [file 1471-2148-8-213-S1.doc]

**Additional file 1.** The references for the mitochondrial sequences included in the datasets used for comparisons to Cuban sequences in the present study.

| **Continent** | **Region** | **Population** | **N** | **References** |
| --- | --- | --- | --- | --- |
| **EUROPE** | South-west | Galicians | 146 | [1-4] |
|  |  | Maragatos | 49 | [5] |
|  |  | León | 60 | [5] |
|  |  | Cantabria | 88 | [6] |
|  |  | Liébana | 71 | [6] |
|  |  | Pasiegos | 80 | [6] |
|  |  | Basques | 173 | [3, 7, 8] |
|  |  | Aragonese | 103 | [9] |
|  |  | Catalans | 78 | [1, 3, 8] |
|  |  | València | 69 | [3, 10] |
|  |  | Central Spain | 89 | [1, 3, 5] |
|  |  | Andalusians | 208 | [1, 3, 5, 11] |
|  |  | Spain | 71 | [8] |
|  |  | North Portugal | 183 | [2, 12] |
|  |  | Central Portugal | 160 | [2, 12] |
|  |  | South Portugal | 194 | [2, 12] |
|  |  | Portugal | 54 | [8] |
|  |  | Mallorca | 45 | [10] |
|  |  | Minorca | 45 | [10] |
|  |  | Eivissa | 50 | [10] |
|  |  | Xuetes | 46 | [10] |
|  |  | Veneto | 68 | [13] |
|  |  | Bologna | 100 | [14] |
|  |  | Tuscans | 49 | [15] |
|  |  | Romans | 48 | [16] |
|  |  | Lazio | 52 | [17] |
|  |  | Abruzzo | 11 | [17] |
|  |  | Central Italians | 81 | [18] |
|  |  | Molise | 62 | [17] |
|  |  | Croatian-Italians (Molise) | 41 | [17] |
|  |  | Campania | 48 | [17] |
|  |  | Puglia | 26 | [17] |
|  |  | Southern Italians | 37 | [19] |
|  |  | Sicilians (OR) | 63 | [19] |
|  |  | Sicilians (Castellammare del Golfo) | 106 | [20] |
|  |  | Alia (Sicily) | 49 | [21] |
|  |  | Sicilians (Troina) | 42 | [16] |
|  |  | Sicilians (Trapani) | 48 | [16] |
|  |  | Corsicans | 46 | [22] |
|  |  | Sardinians | 115 | [16, 23] |
|  | West | Var | 37 | [24] |
|  |  | Périgord-Limousin | 72 | [24] |
|  |  | Brittany | 61 | [24] |
|  |  | Normandy | 39 | [24] |
|  |  | French | 162 | [16, 25, 26] |
|  |  | British | 100 | [27] |
|  |  | Cornish | 92 | [16, 28] |
|  |  | Welsh | 92 | [28] |
|  |  | Irish | 304 | [16, 29] |
|  | Central | South-western Swiss | 154 | [30] |
|  |  | Swiss | 71 | [31] |
|  |  | Bavarians | 49 | [28] |
|  |  | South Germany | 199 | [32] |
|  |  | Münster | 109 | [33] |
|  |  | Rhin-Germans | 50 | [34] |
|  |  | West Pomerania | 299 | [35] |
|  |  | Germans | 174 | [16, 36] |
|  |  | Austrians | 371 | [37, 38] |
|  |  | Poles-Pomerania | 436 | [39] |
|  |  | Poles | 37 | [16] |
|  |  | Czechs | 174 | [16, 40] |
|  | South-east | Slovenians | 231 | [8, 41] |
|  |  | Croatia-Dalmatian coast | 96 | [17] |
|  |  | Hvar (Croatia) | 108 | [42] |
|  |  | Bosnia | 144 | [43] |
|  |  | Romanians Ploiesti | 45 | [44] |
|  |  | Romanians Constanta | 58 | [44] |
|  |  | Romanians | 92 | [16] |
|  |  | Aromuns Romania Kogalniceanu | 42 | [44] |
|  |  | Aromuns Macedonia Stip | 36 | [44] |
|  |  | Aromuns Macedonia Krusevo | 33 | [44] |
|  |  | Aromuns Albania Dukasi | 33 | [44] |
|  |  | Aromuns Albania Andon Poci | 30 | [44] |
|  |  | Macedonians | 35 | [44] |
|  |  | Bulgarians | 141 | [16, 45] |
|  |  | Albanians | 82 | [44, 46] |
|  |  | Sarakatsani | 60 | [16] |
|  |  | Greeks | 90 | [16, 44] |
|  | North | Danes | 37 | [16, 28] |
|  |  | Swedes | 32 | [47] |
|  |  | Norwegians | 305 | [16, 48, 49] |
|  |  | Icelandic | 379 | [28, 50, 51] |
|  |  | Finns | 163 | [16, 28, 51] |
|  |  | Saami | 137 | [51] |
|  |  |  |  |  |
| **Continent** | **Region** | **Population** | **N** | **References** |
| **AMERICA** | Central  (African-Americans) | Colombia (Afro-Colombian; Mercaderes) | 185 | [52] |
|  |  | Dominican Republic | 83 | [53] |
|  | North  (African-Americans) | Gullah/Geecee | 78 | [54] |
|  | South  (African-Americans) | Brazil (Salvador, Portoalegre, etc.) | 42 | [55] |
|  |  | Bahia, Brazil | 80 | [56] |
|  |  | Choco; Columbia | 49 | [57] |
|  |  | Garifunas, Belize | 44 | [57] |
|  |  | Cajueiro; Brazil | 10 | [58] |
|  |  | Cametá; Brazil | 10 | [58] |
|  |  | Ribeirão Preto; Brazil | 20 | [58] |
|  |  | Trombetas; Brazil | 10 | [58] |
|  | Central | Boruca, Amerindian | 7 | [59] |
|  |  | Huetar; Costa Rica | 27 | [60] |
|  |  | Ngobe; Panama | 46 | [61] |
|  |  | Belize; Caribbean | 28 | [62] |
|  |  | Ciboneys; Cuba | 15 | [63] |
|  |  | Emberá; Panamá | 44 | [64] |
|  |  | Quiche; Guatemala | 16 | [65] |
|  |  | Kuna; Panamá | 63 | [66] |
|  |  | Mexico | 87 | [67] |
|  |  | Pima | 40 | [68] |
|  |  | Tainos; Dominican Republic | 19 | [69] |
|  |  | Wounan; Panamá | 31 | [64] |
|  | North | Aleuts; Alaska | 163 | [70] |
|  |  | Apache | 1 | [71] |
|  |  | Athapaskan; Alaska | 21 | [72] |
|  |  | Bella Coola; British Columbia | 40 | [73] |
|  |  | Haida; Queen Charlotte Islands | 41 | [73] |
|  |  | Maya | 3 | [71] |
|  |  | Bella Coola; Canada | 18 | [59] |
|  |  | Yakima; Washington State | 42 | [72] |
|  |  | Apaches | 150 | [74] |
|  |  | Cheyenne | 39 | [68] |
|  |  | Chukchi | 65 | [75] |
|  |  | Eskimos; Siberia | 77 | [75] |
|  |  | Greenland: Eskimo | 82 | [76] |
|  |  | Inupiaq; Alaska | 5 | [72] |
|  |  | Inuit; W. Greenland | 17 | [72] |
|  |  | Inuit; Canada | 129 | [77] |
|  |  | Canada | 100 | [78] |
|  |  | Kwäday Dän Ts'ìnchi remains; Canada | 1 | [79] |
|  |  | Muskogean populations; Creek; Oklahoma; USA | 48 | [80] |
|  |  | North Americans; Asians | 57 | [81] |
|  |  | Northern Ojibwa | 36 | [82] |
|  |  | Chumash | 34 | [83] |
|  |  | North Dakota; North America; Sisseton/Wahpeton Sioux | 109 | [84] |
|  |  | Native North Americans; Sioux | 11 | [85] |
|  |  | USA | 543 | [81] |
|  |  | Navajo; Native North American | 146 | [74] |
|  |  | Vancouver Island; Canada; Nuu-Chah-Nult | 63 | [86] |
|  |  | Norris Farms Oneota | 52 | [87] |
|  | South | Brazil | 3 | [71] |
|  |  | Araucanians; Chile | 45 | [71] |
|  |  | Columbia | 20 | [71] |
|  |  | Mapuches; Argentina | 39 | [88] |
|  |  | Mataco, Amerindian | 13 | [59] |
|  |  | Arsario; Colombia | 28 | [89] |
|  |  | Ayoreo; Amerinds; Bolivia/Paraguay | 91 | [90] |
|  |  | Brazil | 247 | [91] |
|  |  | Equator | 150 | [92] |
|  |  | Prehistoric Andean; Chile | 30 | [93] |
|  |  | Huitoto; Colombia | 64 | [94] |
|  |  | Coyas; Jujui, Salta; Argentina | 61 | [95] |
|  |  | Curiaú; Brazil | 45 | [96] |
|  |  | Gaviao; Indian Arealgarape Lourdes | 27 | [97] |
|  |  | Guarani Kaiowá; Brazil | 120 | [98] |
|  |  | Guarani M´byá; Brazil | 24 | [98] |
|  |  | Guarani Ñandeva; Brazil | 56 | [98] |
|  |  | Guahibo; Venezuela | 59 | [99] |
|  |  | Ignaciano; Bolivia | 15 | [100] |
|  |  | Ijka; Colombia | 31 | [89] |
|  |  | Kogi; Colombia | 21 | [89] |
|  |  | Kaingang-Rio Grande do Sul; Brazil | 57 | [98] |
|  |  | Kaingang-Paraná; Brazil | 21 | [98] |
|  |  | Mapuche; Chile | 34 | [101] |
|  |  | Movima; Llanos de Moxos, Bolivia | 12 | [100] |
|  |  | Cinta Larga | 25 | [102] |
|  |  | Sambaqui do Furinho | 18 | [103] |
|  |  | Selkmam; Argentina, Chile | 24 | [104] |
|  |  | Pehuenche; Chile | 24 | [101] |
|  |  | Pilagá; Formosa; Gran Chaco Argentina | 38 | [105] |
|  |  | Amazonas; Brazil | 92 | [106] |
|  |  | Toba, Chaco; Gran Chaco, Argentina | 67 | [105] |
|  |  | Trinitario; Bolivia | 12 | [100] |
|  |  | Tacuarembó; Uruguay | 24 | [107] |
|  |  | Uruguay | 119 | [108] |
|  |  | Venezuela | 100 | [109] |
|  |  | Wayuú; Colombia | 30 | [89] |
|  |  | Wichí o Mataco, Chaco; Gran Chaco, Argentina | 99 | [105] |
|  |  | Xavante; Rio das Mortes; Brazil | 25 | [97] |
|  |  | Yaghan; Chile | 15 | [101] |
|  |  | Yanomami; Venezuela/Brazil | 129 | [110] |
|  |  | Yanomami; Venezuela | 155 | [111] |
|  |  | Yuracare, Llanos de Moxos; Bolivia | 15 | [100] |
|  |  | Zoro; Brazil | 30 | [97] |
|  |  |  |  |  |
|  |  |  |  |  |
|  |  |  |  |  |
|  |  |  |  |  |
|  |  |  |  |  |
|  |  |  |  |  |
|  |  |  |  |  |
|  |  |  |  |  |
|  |  |  |  |  |
|  |  |  |  |  |
| **Continent** | **Region** | **Population** | **N** | **References** |
| **AFRICA** |  | Makamba; Africa | 53 | [112] |
|  |  | Africa | 2 | [113] |
|  |  | Africa | 1 | [113] |
|  |  | Africa | 1 | [113] |
|  |  | Africa | 3 | [113] |
|  | East | Urban Nairobi; Kenya | 100 | [114] |
|  |  | Kenya | 100 | [78] |
|  |  | Yemen | 115 | [115] |
|  |  | Dakota; Tanzania | 18 | [116] |
|  |  | Nubia; S. Egypt/N. Sudan | 80 | [117] |
|  |  | Southern Sudan | 76 | [117] |
|  |  | Ethiopia | 25 | [118] |
|  |  | Ethiopia | 171 | [119] |
|  |  | Ethiopia | 74 | [120] |
|  |  | Hadza; Tanzania | 61 | [121] |
|  |  | Kikuyu; Kenya | 24 | [122] |
|  |  | Somalia | 27 | [122] |
|  |  | Turkana; Kenya | 37 | [122] |
|  | North | Morocco, Souss Valley | 50 | [123] |
|  |  | Azores | 179 | [124] |
|  |  | Madeira | 155 | [124] |
|  |  | Ghardaia, Algeria | 86 | [8] |
|  |  | Berbers; Chenini; Tunisia | 53 | [125] |
|  |  | Berbers, Matmata; Tunisia | 49 | [125] |
|  |  | Berbers; Sened; Tunisia | 53 | [125] |
|  |  | Egypt | 68 | [117] |
|  |  | Egypt | 4839 | [126] |
|  |  | Algeria | 47 | [3] |
|  |  | Arabs; Morocco | 18 | [3] |
|  |  | Berbers; Morocco | 64 | [3] |
|  |  | Saharawi | 56 | [3] |
|  |  | Tunisians | 47 | [3] |
|  |  | Canary Islands | 300 | [127] |
|  |  | Berber speaker; Morocco | 64 | [128] |
|  |  | Mauritanian | 30 | [128] |
|  |  | non-Berber speaker; Morocco | 32 | [128] |
|  |  | West Saharan | 25 | [128] |
|  | South | !Kung; South Africa | 43 | [129] |
|  |  | Khwe; South Africa | 31 | [129] |
|  |  | South Africa | 2 | [130] |
|  |  | Dama; Namibia | 21 | [130] |
|  |  | Herero; Namibia | 12 | [130] |
|  |  | Nama; Namibia | 18 | [130] |
|  |  | Sekele !Kung | 38 | [130] |
|  |  | Sotho; South Africa | 20 | [130] |
|  |  | Xhosa; South Africa | 8 | [130] |
|  |  | Zulu; South Africa | 8 | [130] |
|  |  | Botswana | 15 | [131] |
|  |  | Herero; Namibia | 27 | [121] |
|  |  | !Kung; Botswana | 25 | [121] |
|  | South-east | Mozambique | 109 | [132] |
|  |  | Mozambique | 307 | [133] |
|  | South-west | Cabinda | 110 | [134] |
|  |  | Mbundu; Angola | 44 | [135] |
|  | West | Cape Verde | 292 | [136] |
|  |  | Fulani Banfora; Burkina Faso | 50 | [137] |
|  |  | Fulani Tindangou; Burkina Faso | 47 | [137] |
|  |  | Bambara; Mali | 19 | [54] |
|  |  | Malinke; Mali | 61 | [54] |
|  |  | Bambara; Mali | 124 | [138] |
|  |  | Mauritania | 64 | [138] |
|  |  | Senegal: Mandenka | 119 | [139] |
|  |  | Limba; Sierra Leona | 68 | [140] |
|  |  | Loko; Sierra Leona | 29 | [140] |
|  |  | Mende; Sierra Leona | 59 | [140] |
|  |  | Temne; Sierra Leona | 121 | [140] |
|  |  | Senegal | 50 | [128] |
|  |  | Serer; Senegal | 23 | [128] |
|  |  | Wolof; Senegal | 48 | [128] |
|  |  | Lubumbashi city; Shaba province; Cameroon | 10 | [58] |
|  |  | Yoruba; Nigeria | 35 | [121] |
|  |  | Fulbe; Nigeria | 60 | [122] |
|  |  | Niger Hausa; Nigeria | 20 | [122] |
|  |  | Kanuri; Chad | 14 | [122] |
|  |  | Niger Songhai | 10 | [122] |
|  |  | Niger; Mali; Algeria; Tuareg | 23 | [122] |
|  |  | Yoruba; Nigeria | 35 | [122] |
|  | West-central | Arabs Chad; Chad | 27 | [141] |
|  |  | Buduma; Lake Chad | 30 | [141] |
|  |  | Fali; Cameroon | 40 | [141] |
|  |  | Fulani Bongor; Chad | 49 | [141] |
|  |  | Fulani Tcheboua; Cameroon | 40 | [141] |
|  |  | Hide; Northern Cameroon and South-eastern Niger | 23 | [141] |
|  |  | Kanuri; Borno; Nigeria | 31 | [141] |
|  |  | Kanenbu; Kanem Chad | 50 | [141] |
|  |  | Kotoco; Northern Cameroon and South-eastern Niger | 56 | [141] |
|  |  | Mafa; Northern Cameroon and South-eastern Niger | 32 | [141] |
|  |  | Masa; Northern Cameroon and South-eastern Niger | 32 | [141] |
|  |  | Arabs Shuwa; Borno state; Nigeria | 38 | [141] |
|  |  | Bakaka; southern Cameroon | 50 | [142] |
|  |  | Bamileke; southern Cameroon | 48 | [142] |
|  |  | Basa; South Cameroon | 46 | [142] |
|  |  | Daba; northern Cameroon | 20 | [142] |
|  |  | Ewondo; southern Cameroon | 53 | [142] |
|  |  | Fali; northern Cameroon | 41 | [142] |
|  |  | Fulbe; northern Cameroon | 34 | [142] |
|  |  | Mandara; North Cameroon | 37 | [142] |
|  |  | Ouldeme; northern Cameroon | 28 | [142] |
|  |  | Podokwo; North Cameroon | 39 | [142] |
|  |  | Tali; northern Cameroon | 20 | [142] |
|  |  | Tupuri; North Cameroon | 25 | [142] |
|  |  | Mbenzele-Pygmy; Central African Replublic (CAR) | 57 | [143] |
|  |  | São Tomé and Principe; Equatorial Guinea | 50 | [144] |
|  |  | Fang; Equitorial Guinea | 11 | [145] |
|  |  | Yaoundé city; Congo | 10 | [58] |
|  |  | Forros, São Tomé and Principe; Equatorial Guinea | 35 | [146] |
|  |  | Biaka; Central African Republic (CAR) | 17 | [121] |
|  |  | Mbuti; Zaire | 20 | [121] |

**References**

1. Crespillo M, Luque JA, Paredes M, Fernandez R, Ramirez E, Valverde JL: **Mitochondrial DNA sequences for 118 individuals from northeastern Spain**. *International journal of legal medicine* 2000, **114**(1-2):130-132.

2. Gonzalez AM, Brehm A, Perez JA, Maca-Meyer N, Flores C, Cabrera VM: **Mitochondrial DNA affinities at the Atlantic fringe of Europe**. *American journal of physical anthropology* 2003, **120**(4):391-404.

3. Plaza S, Calafell F, Helal A, Bouzerna N, Lefranc G, Bertranpetit J, Comas D: **Joining the pillars of Hercules: mtDNA sequences show multidirectional gene flow in the western Mediterranean**. *Annals of human genetics* 2003, **67**(Pt 4):312-328.

4. Salas A, Comas D, Lareu MV, Bertranpetit J, Carracedo A: **mtDNA analysis of the Galician population: a genetic edge of European variation**. *Eur J Hum Genet* 1998, **6**(4):365-375.

5. Larruga JM, Diez F, Pinto FM, Flores C, Gonzalez AM: **Mitochondrial DNA characterisation of European isolates: the Maragatos from Spain**. *Eur J Hum Genet* 2001, **9**(9):708-716.

6. Maca-Meyer N, Sanchez-Velasco P, Flores C, Larruga JM, Gonzalez AM, Oterino A, Leyva-Cobian F: **Y chromosome and mitochondrial DNA characterization of Pasiegos, a human isolate from Cantabria (Spain)**. *Annals of human genetics* 2003, **67**(Pt 4):329-339.

7. Bertranpetit J, Sala J, Calafell F, Underhill PA, Moral P, Comas D: **Human mitochondrial DNA variation and the origin of Basques**. *Annals of human genetics* 1995, **59**(Pt 1):63-81.

8. Corte-Real HB, Macaulay VA, Richards MB, Hariti G, Issad MS, Cambon-Thomsen A, Papiha S, Bertranpetit J, Sykes BC: **Genetic diversity in the Iberian Peninsula determined from mitochondrial sequence analysis**. *Annals of human genetics* 1996, **60**(Pt 4):331-350.

9. Martinez-Jarreta B, Prades A, Calafell F, Budowle B: **Mitochondrial DNA HVI and HVII variation in a north-east Spanish population**. *Journal of forensic sciences* 2000, **45**(5):1162-1163.

10. Picornell A, Gomez-Barbeito L, Tomas C, Castro JA, Ramon MM: **Mitochondrial DNA HVRI variation in Balearic populations**. *American journal of physical anthropology* 2005, **128**(1):119-130.

11. López-Soto M, Sanz P: **Polimorfismos de ADN Mitocondrial en individuos residentes en Andalucia y Extremadura**. *Cuadernos de Medicina Forense* 2000, **20**:17-24.

12. Pereira L, Prata MJ, Amorim A: **Diversity of mtDNA lineages in Portugal: not a genetic edge of European variation**. *Annals of human genetics* 2000, **64**(Pt 6):491-506.

13. Mogentale-Profizi N, Chollet L, Stevanovitch A, Dubut V, Poggi C, Pradie MP, Spadoni JL, Gilles A, Beraud-Colomb E: **Mitochondrial DNA sequence diversity in two groups of Italian Veneto speakers from Veneto**. *Annals of human genetics* 2001, **65**(Pt 2):153-166.

14. Bini C, Ceccardi S, Luiselli D, Ferri G, Pelotti S, Colalongo C, Falconi M, Pappalardo G: **Different informativeness of the three hypervariable mitochondrial DNA regions in the population of Bologna (Italy)**. *Forensic Sci Int* 2003, **135**(1):48-52.

15. Francalacci P, Bertranpetit J, Calafell F, Underhill PA: **Sequence diversity of the control region of mitochondrial DNA in Tuscany and its implications for the peopling of Europe**. *American journal of physical anthropology* 1996, **100**(4):443-460.

16. Richards M, Macaulay V, Hickey E, Vega E, Sykes B, Guida V, Rengo C, Sellitto D, Cruciani F, Kivisild T *et al*: **Tracing European founder lineages in the Near Eastern mtDNA pool**. *American journal of human genetics* 2000, **67**(5):1251-1276.

17. Babalini C, Martinez-Labarga C, Tolk HV, Kivisild T, Giampaolo R, Tarsi T, Contini I, Barac L, Janicijevic B, Martinovic Klaric I *et al*: **The population history of the Croatian linguistic minority of Molise (southern Italy): a maternal view**. *Eur J Hum Genet* 2005, **13**(8):902-912.

18. Tagliabracci A, Turchi C, Buscemi L, Sassaroli C: **Polymorphism of the mitochondrial DNA control region in Italians**. *International journal of legal medicine* 2001, **114**(4-5):224-228.

19. Rickards O, Martínez labarga C, Casalotti R, Castellana G, Tunzi sisto AM, Mallegni F: **MtDNA variability in extinct and extant populations of Sicily and southern Italy**. In: *Archaeogenetics: DNA and the population prehistory of Europe.* Edited by K.Boyle CR, vol. McDonald Institute Monographs. Cambridge: McDonald Institute for Archaeological Research; 2000: 175-183.

20. Cali F, Le Roux MG, D'Anna R, Flugy A, De Leo G, Chiavetta V, Ayala GF, Romano V: **MtDNA control region and RFLP data for Sicily and France**. *International journal of legal medicine* 2001, **114**(4-5):229-231.

21. Vona G, Ghiani ME, Calo CM, Vacca L, Memmi M, Varesi L: **Mitochondrial DNA sequence analysis in Sicily**. *Am J Hum Biol* 2001, **13**(5):576-589.

22. Varesi L, Memmi M, Cristofari MC, Mameli GE, Calo CM, Vona G: **Mitochondrial control-region sequence variation in the Corsican population, France**. *Am J Hum Biol* 2000, **12**(3):339-351.

23. Di Rienzo A, Wilson AC: **Branching pattern in the evolutionary tree for human mitochondrial DNA**. *Proc Natl Acad Sci U S A* 1991, **88**(5):1597-1601.

24. Dubut V, Chollet L, Murail P, Cartault F, Beraud-Colomb E, Serre M, Mogentale-Profizi N: **mtDNA polymorphisms in five French groups: importance of regional sampling**. *Eur J Hum Genet* 2004, **12**(4):293-300.

25. Danan C, Sternberg D, Van Steirteghem A, Cazeneuve C, Duquesnoy P, Besmond C, Goossens M, Lissens W, Amselem S: **Evaluation of parental mitochondrial inheritance in neonates born after intracytoplasmic sperm injection**. *American journal of human genetics* 1999, **65**(2):463-473.

26. Rousselet F, Mangin P: **Mitochondrial DNA polymorphisms: a study of 50 French Caucasian individuals and application to forensic casework**. *International journal of legal medicine* 1998, **111**(6):292-298.

27. Piercy R, Sullivan KM, Benson N, Gill P: **The application of mitochondrial DNA typing to the study of white Caucasian genetic identification**. *International journal of legal medicine* 1993, **106**(2):85-90.

28. Richards M, Corte-Real H, Forster P, Macaulay V, Wilkinson-Herbots H, Demaine A, Papiha S, Hedges R, Bandelt HJ, Sykes B: **Paleolithic and neolithic lineages in the European mitochondrial gene pool**. *American journal of human genetics* 1996, **59**(1):185-203.

29. McEvoy B, Richards M, Forster P, Bradley DG: **The Longue Duree of genetic ancestry: multiple genetic marker systems and Celtic origins on the Atlantic facade of Europe**. *American journal of human genetics* 2004, **75**(4):693-702.

30. Dimo-Simonin N, Grange F, Taroni F, Brandt-Casadevall C, Mangin P: **Forensic evaluation of mtDNA in a population from south west Switzerland**. *International journal of legal medicine* 2000, **113**(2):89-97.

31. Pult I, Sajantila A, Simanainen J, Georgiev O, Schaffner W, Paabo S: **Mitochondrial DNA sequences from Switzerland reveal striking homogeneity of European populations**. *Biol Chem Hoppe Seyler* 1994, **375**(12):837-840.

32. Lutz S, Weisser HJ, Heizmann J, Pollak S: **Location and frequency of polymorphic positions in the mtDNA control region of individuals from Germany**. *International journal of legal medicine* 1998, **111**(2):67-77.

33. Pfeiffer H, Brinkmann B, Huhne J, Rolf B, Morris AA, Steighner R, Holland MM, Forster P: **Expanding the forensic German mitochondrial DNA control region database: genetic diversity as a function of sample size and microgeography**. *International journal of legal medicine* 1999, **112**(5):291-298.

34. Baasner A, Schafer C, Junge A, Madea B: **Polymorphic sites in human mitochondrial DNA control region sequences: population data and maternal inheritance**. *Forensic Sci Int* 1998, **98**(3):169-178.

35. Poetsch M, Wittig H, Krause D, Lignitz E: **Mitochondrial diversity of a northeast German population sample**. *Forensic Sci Int* 2003, **137**(2-3):125-132.

36. Hofmann S, Jaksch M, Bezold R, Mertens S, Aholt S, Paprotta A, Gerbitz KD: **Population genetics and disease susceptibility: characterization of central European haplogroups by mtDNA gene mutations, correlation with D loop variants and association with disease**. *Hum Mol Genet* 1997, **6**(11):1835-1846.

37. Brandstatter A, Niederstatter H, Pavlic M, Grubwieser P, Parson W: **Generating population data for the EMPOP database - an overview of the mtDNA sequencing and data evaluation processes considering 273 Austrian control region sequences as example**. *Forensic Sci Int* 2007, **166**(2-3):164-175.

38. Parson W, Parsons TJ, Scheithauer R, Holland MM: **Population data for 101 Austrian Caucasian mitochondrial DNA d-loop sequences: application of mtDNA sequence analysis to a forensic case**. *International journal of legal medicine* 1998, **111**(3):124-132.

39. Malyarchuk BA, Grzybowski T, Derenko MV, Czarny J, Wozniak M, Miscicka-Sliwka D: **Mitochondrial DNA variability in Poles and Russians**. *Annals of human genetics* 2002, **66**(Pt 4):261-283.

40. Vanecek T, Vorel F, Sip M: **Mitochondrial DNA D-loop hypervariable regions: Czech population data**. *International journal of legal medicine* 2004, **118**(1):14-18.

41. Zupanic Pajnic I, Balazic J, Komel R: **Sequence polymorphism of the mitochondrial DNA control region in the Slovenian population**. *International journal of legal medicine* 2004, **118**(1):1-4.

42. Tolk HV, Barac L, Pericic M, Klaric IM, Janicijevic B, Campbell H, Rudan I, Kivisild T, Villems R, Rudan P: **The evidence of mtDNA haplogroup F in a European population and its ethnohistoric implications**. *Eur J Hum Genet* 2001, **9**(9):717-723.

43. Malyarchuk BA, Grzybowski T, Derenko MV, Czarny J, Drobnic K, Miscicka-Sliwka D: **Mitochondrial DNA variability in Bosnians and Slovenians**. *Annals of human genetics* 2003, **67**(Pt 5):412-425.

44. Bosch E, Calafell F, Gonzalez-Neira A, Flaiz C, Mateu E, Scheil HG, Huckenbeck W, Efremovska L, Mikerezi I, Xirotiris N *et al*: **Paternal and maternal lineages in the Balkans show a homogeneous landscape over linguistic barriers, except for the isolated Aromuns**. *Annals of human genetics* 2006, **70**(Pt 4):459-487.

45. Calafell F, Underhill P, Tolun A, Angelicheva D, Kalaydjieva L: **From Asia to Europe: mitochondrial DNA sequence variability in Bulgarians and Turks**. *Annals of human genetics* 1996, **60**(Pt 1):35-49.

46. Belledi M, Poloni ES, Casalotti R, Conterio F, Mikerezi I, Tagliavini J, Excoffier L: **Maternal and paternal lineages in Albania and the genetic structure of Indo-European populations**. *Eur J Hum Genet* 2000, **8**(7):480-486.

47. Sajantila A, Salem AH, Savolainen P, Bauer K, Gierig C, Paabo S: **Paternal and maternal DNA lineages reveal a bottleneck in the founding of the Finnish population**. *Proc Natl Acad Sci U S A* 1996, **93**(21):12035-12039.

48. Opdal SH, Rognum TO, Vege A, Stave AK, Dupuy BM, Egeland T: **Increased number of substitutions in the D-loop of mitochondrial DNA in the sudden infant death syndrome**. *Acta Paediatr* 1998, **87**(10):1039-1044.

49. Passarino G, Cavalleri GL, Lin AA, Cavalli-Sforza LL, Borresen-Dale AL, Underhill PA: **Different genetic components in the Norwegian population revealed by the analysis of mtDNA and Y chromosome polymorphisms**. *Eur J Hum Genet* 2002, **10**(9):521-529.

50. Helgason A, Sigureth ardottir S, Gulcher JR, Ward R, Stefansson K: **mtDNA and the origin of the Icelanders: deciphering signals of recent population history**. *American journal of human genetics* 2000, **66**(3):999-1016.

51. Sajantila A, Lahermo P, Anttinen T, Lukka M, Sistonen P, Savontaus ML, Aula P, Beckman L, Tranebjaerg L, Gedde-Dahl T *et al*: **Genes and languages in Europe: an analysis of mitochondrial lineages**. *Genome Res* 1995, **5**(1):42-52.

52. Salas A, Acosta A, Álvarez-Iglesias V, Phillips C, Lareu MV, Carracedo A: **The mtDNA Ancestry of Admixed Colombian Populations**. *American journal of human biology* 2008, **in press**.

53. Tajima A, Hamaguchi K, Terao H, Oribe A, Perrotta VM, Baez CA, Arias JR, Yoshimatsu H, Sakata T, Horai S: **Genetic background of people in the Dominican Republic with or without obese type 2 diabetes revealed by mitochondrial DNA polymorphism**. *J Hum Genet* 2004, **49**(9):495-499.

54. Ely B, Wilson JL, Jackson F, Jackson BA: **African-American mitochondrial DNAs often match mtDNAs found in multiple African ethnic groups**. *BMC Biol* 2006, **4**:34.

55. Bortolini MC, Zago MA, Salzano FM, Silva-Junior WA, Bonatto SL, da Silva MC, Weimer TA: **Evolutionary and anthropological implications of mitochondrial DNA variation in African Brazilian populations**. *Human biology; an international record of research* 1997, **69**(2):141-159.

56. Santos MVea. In: *VII Jornadas de Genética Forense.* Barcelona, Spain; 2002.

57. Salas A, Richards M, Lareu MV, Sobrino B, Silva S, Matamoros M, Macaulay V, Carracedo A: **Shipwrecks and founder effects: divergent demographic histories reflected in Caribbean mtDNA**. *American journal of physical anthropology* 2005, **128**(4):855-860.

58. Silva WA, Bortolini MC, Schneider MP, Marrero A, Elion J, Krishnamoorthy R, Zago MA: **MtDNA haplogroup analysis of black Brazilian and sub-Saharan populations: implications for the Atlantic slave trade**. *Human biology; an international record of research* 2006, **78**(1):29-41.

59. Torroni A, Schurr TG, Cabell MF, Brown MD, Neel JV, Larsen M, Smith DG, Vullo CM, Wallace DC: **Asian affinities and continental radiation of the four founding Native American mtDNAs**. *American journal of human genetics* 1993, **53**(3):563-590.

60. Santos M, Ward RH, Barrantes R: **mtDNA variation in the Chibcha Amerindian Huetar from Costa Rica**. *Human biology; an international record of research* 1994, **66**(6):963-977.

61. Kolman CJ, Bermingham E, Cooke R, Ward RH, Arias TD, Guionneau-Sinclair F: **Reduced mtDNA diversity in the Ngobe Amerinds of Panama**. *Genetics* 1995, **140**(1):275-283.

62. Monsalve MV, Hagelberg E: **Mitochondrial DNA polymorphisms in Carib people of Belize**. *Proc Biol Sci* 1997, **264**(1385):1217-1224.

63. Lalueza-Fox C, Gilbert MT, Martínez-Fuentes AJ, Calafell F, Bertranpetit J: **Mitochondrial DNA from pre-Columbian Ciboneys from Cuba and the prehistoric colonization of the Caribbean**. *American journal of physical anthropology* 2003, **121**(2):97-108.

64. Kolman CJ, Bermingham E: **Mitochondrial and nuclear DNA diversity in the Choco and Chibcha Amerinds of Panama**. *Genetics* 1997, **147**(3):1289-1302.

65. Boles TC, Snow CC, Stover E: **Forensic DNA testing on skeletal remains from mass graves: a pilot project in Guatemala**. *Journal of forensic sciences* 1995, **40**(3):349-355.

66. Batista O, Kolman CJ, Bermingham E: **Mitochondrial DNA diversity in the Kuna Amerinds of Panama**. *Hum Mol Genet* 1995, **4**(5):921-929.

67. Green LD, Derr JN, Knight A: **mtDNA affinities of the peoples of North-Central Mexico**. *American journal of human genetics* 2000, **66**(3):989-998.

68. Kittles RA, Bergen AW, Urbanek M, Virkkunen M, Linnoila M, Goldman D, Long JC: **Autosomal, mitochondrial, and Y chromosome DNA variation in Finland: evidence for a male-specific bottleneck**. *American journal of physical anthropology* 1999, **108**(4):381-399.

69. Lalueza-Fox C, Calderon FL, Calafell F, Morera B, Bertranpetit J: **MtDNA from extinct Tainos and the peopling of the Caribbean**. *Annals of human genetics* 2001, **65**(Pt 2):137-151.

70. Rubicz R, Schurr TG, Babb PL, Crawford MH: **Mitochondrial DNA variation and the origins of the Aleuts**. *Human biology; an international record of research* 2003, **75**(6):809-835.

71. Horai S, Kondo R, Nakagawa-Hattori Y, Hayashi S, Sonoda S, Tajima K: **Peopling of the Americas, founded by four major lineages of mitochondrial DNA**. *Molecular biology and evolution* 1993, **10**(1):23-47.

72. Shields GF, Schmiechen AM, Frazier BL, Redd A, Voevoda MI, Reed JK, Ward RH: **mtDNA sequences suggest a recent evolutionary divergence for Beringian and northern North American populations**. *American journal of human genetics* 1993, **53**(3):549-562.

73. Ward RH, Redd A, Valencia D, Frazier B, Paabo S: **Genetic and linguistic differentiation in the Americas**. *Proc Natl Acad Sci U S A* 1993, **90**(22):10663-10667.

74. Budowle B, Allard MW, Fisher CL, Isenberg AR, Monson KL, Stewart JE, Wilson MR, Miller KW: **HVI and HVII mitochondrial DNA data in Apaches and Navajos**. *International journal of legal medicine* 2002, **116**(4):212-215.

75. Starikovskaya YB, Sukernik RI, Schurr TG, Kogelnik AM, Wallace DC: **mtDNA diversity in Chukchi and Siberian Eskimos: implications for the genetic history of Ancient Beringia and the peopling of the New World**. *American journal of human genetics* 1998, **63**(5):1473-1491.

76. Saillard J, Forster P, Lynnerup N, Bandelt HJ, Norby S: **mtDNA variation among Greenland Eskimos: the edge of the Beringian expansion**. *American journal of human genetics* 2000, **67**(3):718-726.

77. Meyer S, Weiss G, von Haeseler A: **Pattern of nucleotide substitution and rate heterogeneity in the hypervariable regions I and II of human mtDNA**. *Genetics* 1999, **152**(3):1103-1110.

78. Horai S, Hayasaka K: **Intraspecific nucleotide sequence differences in the major noncoding region of human mitochondrial DNA**. *American journal of human genetics* 1990, **46**(4):828-842.

79. Monsalve MV, Stone AC, Lewis CM, Rempel A, Richards M, Straathof D, Devine DV: **Brief communication: molecular analysis of the Kwaday Dan Ts'finchi ancient remains found in a glacier in Canada**. *American journal of physical anthropology* 2002, **119**(3):288-291.

80. Bolnick DA, Smith DG: **Unexpected patterns of mitochondrial DNA variation among Native Americans from the southeastern United States**. *American journal of physical anthropology* 2003, **122**(4):336-354.

81. (SWGDAM) SwgoDam: **mtDNA population database**. In*.*: Federal Bureau of Investigation (FBI).

82. Brown MD, Hosseini SH, Torroni A, Bandelt HJ, Allen JC, Schurr TG, Scozzari R, Cruciani F, Wallace DC: **mtDNA haplogroup X: An ancient link between Europe/Western Asia and North America?** *American journal of human genetics* 1998, **63**(6):1852-1861.

83. Lorenz JG, Smith DG: **Distribution of sequence variation in the mtDNA control region of Native North Americans**. *Human biology; an international record of research* 1997, **69**(6):749-776.

84. Malhi RS, Schultz BA, Smith DG: **Distribution of mitochondrial DNA lineages among Native American tribes of Northeastern North America**. *Human biology; an international record of research* 2001, **73**(1):17-55.

85. Smith DG, Malhi RS, Eshleman J, Lorenz JG, Kaestle FA: **Distribution of mtDNA haplogroup X among Native North Americans**. *American journal of physical anthropology* 1999, **110**(3):271-284.

86. Ward RH, Frazier BL, Dew-Jager K, Paabo S: **Extensive mitochondrial diversity within a single Amerindian tribe**. *Proc Natl Acad Sci U S A* 1991, **88**(19):8720-8724.

87. Stone AC, Stoneking M: **mtDNA analysis of a prehistoric Oneota population: implications for the peopling of the New World**. *American journal of human genetics* 1998, **62**(5):1153-1170.

88. Ginther C, Corach D, Penacino GA, Rey JA, Carnese FR, Hutz MH, Anderson A, Just J, Salzano FM, King MC: **Genetic variation among the Mapuche Indians from the Patagonian region of Argentina: mitochondrial DNA sequence variation and allele frequencies of several nuclear genes**. *Exs* 1993, **67**:211-219.

89. Melton PE, Briceno I, Gomez A, Devor EJ, Bernal JE, Crawford MH: **Biological relationship between Central and South American Chibchan speaking populations: evidence from mtDNA**. *American journal of physical anthropology* 2007, **133**(1):753-770.

90. Dornelles CL, Battilana J, Fagundes NJ, Freitas LB, Bonatto SL, Salzano FM: **Mitochondrial DNA and Alu insertions in a genetically peculiar population: the Ayoreo Indians of Bolivia and Paraguay**. *Am J Hum Biol* 2004, **16**(4):479-488.

91. Alves-Silva J, da Silva Santos M, Guimaraes PE, Ferreira AC, Bandelt HJ, Pena SD, Prado VF: **The ancestry of Brazilian mtDNA lineages**. *American journal of human genetics* 2000, **67**(2):444-461.

92. Rickards O, Martinez-Labarga C, Lum JK, De Stefano GF, Cann RL: **mtDNA history of the Cayapa Amerinds of Ecuador: detection of additional founding lineages for the Native American populations**. *American journal of human genetics* 1999, **65**(2):519-530.

93. Moraga M, Santoro CM, Standen VG, Carvallo P, Rothhammer F: **Microevolution in prehistoric Andean populations: chronologic mtDNA variation in the desert valleys of northern Chile**. *American journal of physical anthropology* 2005, **127**(2):170-181.

94. Torres MM, Bravi CM, Bortolini MC, Duque C, Callegari-Jacques S, Ortiz D, Bedoya G, Groot de Restrepo H, Ruiz-Linares A: **A revertant of the major founder Native American haplogroup C common in populations from northern South America**. *Am J Hum Biol* 2006, **18**(1):59-65.

95. Alvarez-Iglesias V, Jaime J, Carracedo A, Salas A: **Coding region mitochondrial DNA SNPs: Targeting East Asian and Native American haplogroups**. *Forensic Science International: Genetics* 2007, **1**(1):44-45.

96. Ribeiro-dos-Santos AK, Pereira JM, Lobato MR, Carvalho BM, Guerreiro JF, Batista Dos Santos SE: **Dissimilarities in the process of formation of Curiau, a semi-isolated Afro-Brazilian population of the Amazon region**. *Am J Hum Biol* 2002, **14**(4):440-447.

97. Ward RH, Salzano FM, Bonatto SL, Hutz MH, Coimbra CEA, Santos RV: **Mitochondrial DNA Polymorphism in three Brazilian Indian Tribes**. *American journal of human biology* 1996, **8**(3):317-323.

98. Marrero AR, Das Neves Leite FP, De Almeida Carvalho B, Peres LM, Kommers TC, Da Cruz IM, Salzano FM, Ruiz-Linares A, Da Silva Junior WA, Bortolini MC: **Heterogeneity of the genome ancestry of individuals classified as White in the state of Rio Grande do Sul, Brazil**. *Am J Hum Biol* 2005, **17**(4):496-506.

99. Vona G, Falchi A, Moral P, Calo CM, Varesi L: **Mitochondrial sequence variation in the Guahibo Amerindian population from Venezuela**. *American journal of physical anthropology* 2005, **127**(3):361-369.

100. Bert F, Corella A, Gene M, Perez-Perez A, Turbon D: **Mitochondrial DNA diversity in the Llanos de Moxos: Moxo, Movima and Yuracare Amerindian populations from Bolivia lowlands**. *Annals of human biology* 2004, **31**(1):9-28.

101. Moraga ML, Rocco P, Miquel JF, Nervi F, Llop E, Chakraborty R, Rothhammer F, Carvallo P: **Mitochondrial DNA polymorphisms in Chilean aboriginal populations: implications for the peopling of the southern cone of the continent**. *American journal of physical anthropology* 2000, **113**(1):19-29.

102. Dornelles CL, Bonatto SL, De Freitas LB, Salzano FM: **Is haplogroup X present in extant South American Indians?** *American journal of physical anthropology* 2005, **127**(4):439-448.

103. Ribeiro-dos-Santos AK, Santos SE, Machado AL, Guapindaia V, Zago MA: **Heterogeneity of mitochondrial DNA haplotypes in Pre-Columbian Natives of the Amazon region**. *American journal of physical anthropology* 1996, **101**(1):29-37.

104. Garcia-Bour J, Perez-Perez A, Alvarez S, Fernandez E, Lopez-Parra AM, Arroyo-Pardo E, Turbon D: **Early population differentiation in extinct aborigines from Tierra del Fuego-Patagonia: ancient mtDNA sequences and Y-chromosome STR characterization**. *American journal of physical anthropology* 2004, **123**(4):361-370.

105. Cabana GS, Merriwether DA, Hunley K, Demarchi DA: **Is the genetic structure of Gran Chaco populations unique? Interregional perspectives on native South American mitochondrial DNA variation**. *American journal of physical anthropology* 2006, **131**(1):108-119.

106. Santos SE, Ribeiro-Dos-Santos AK, Meyer D, Zago MA: **Multiple founder haplotypes of mitochondrial DNA in Amerindians revealed by RFLP and sequencing**. *Annals of human genetics* 1996, **60**(Pt 4):305-319.

107. Bonilla C, Bertoni B, Gonzalez S, Cardoso H, Brum-Zorrilla N, Sans M: **Substantial Native American female contribution to the population of Tacuarembo, Uruguay, reveals past episodes of sex-biased gene flow**. *Am J Hum Biol* 2004, **16**(3):289-297.

108. Pagano S, Sans M, Pimenoff V, Cantera AM, Alvarez JC, Lorente JA, Peco JM, Mones P, Sajantila A: **Assessment of HV1 and HV2 mtDNA variation for forensic purposes in an Uruguayan population sample**. *Journal of forensic sciences* 2005, **50**(5):1239-1242.

109. Lander N, Rojas MG, Chiurillo MA, Ramírez JL: **Haplotype diversity in Human mitochondrial DNA hypervariable regions I, II and III in the city of Caracas (Venezuela)**. *Forensic Sci Int Genet* 2008, **in press**.

110. Merriwether DA, Kemp BM, Crews DE, Neel JV: **Gene flow and genetic variation in the Yanomama as revealed by mitochondrial DNA**. In: *America Past, America Present: Genes and Languages in the Americas and Beyond.* Edited by C R. Cambridge: McDonald Institute for Archaeological Research; 2000: 89-124.

111. Williams SR, Chagnon NA, Spielman RS: **Nuclear and mitochondrial genetic variation in the Yanomamo: a test case for ancient DNA studies of prehistoric populations**. *American journal of physical anthropology* 2002, **117**(3):246-259.

112. Ingman M, Kaessmann H, Paabo S, Gyllensten U: **Mitochondrial genome variation and the origin of modern humans**. *Nature* 2000, **408**(6813):708-713.

113. Watson E, Bauer K, Aman R, Weiss G, von Haeseler A, Paabo S: **mtDNA sequence diversity in Africa**. *American journal of human genetics* 1996, **59**(2):437-444.

114. Brandstatter A, Peterson CT, Irwin JA, Mpoke S, Koech DK, Parson W, Parsons TJ: **Mitochondrial DNA control region sequences from Nairobi (Kenya): inferring phylogenetic parameters for the establishment of a forensic database**. *International journal of legal medicine* 2004, **118**(5):294-306.

115. Kivisild T, Reidla M, Metspalu E, Rosa A, Brehm A, Pennarun E, Parik J, Geberhiwot T, Usanga E, Villems R: **Ethiopian mitochondrial DNA heritage: tracking gene flow across and around the gate of tears**. *American journal of human genetics* 2004, **75**(5):752-770.

116. Knight A, Underhill PA, Mortensen HM, Zhivotovsky LA, Lin AA, Henn BM, Louis D, Ruhlen M, Mountain JL: **African Y chromosome and mtDNA divergence provides insight into the history of click languages**. *Curr Biol* 2003, **13**(6):464-473.

117. Krings M, Salem AE, Bauer K, Geisert H, Malek AK, Chaix L, Simon C, Welsby D, Di Rienzo A, Utermann G *et al*: **mtDNA analysis of Nile River Valley populations: A genetic corridor or a barrier to migration?** *American journal of human genetics* 1999, **64**(4):1166-1176.

118. Quintana-Murci L, Semino O, Bandelt HJ, Passarino G, McElreavey K, Santachiara-Benerecetti AS: **Genetic evidence of an early exit of Homo sapiens sapiens from Africa through eastern Africa**. *Nature genetics* 1999, **23**(4):437-441.

119. Reidla M, Kivisild T, Metspalu E, Kaldma K, Tambets K, Tolk HV, Parik J, Loogvali EL, Derenko M, Malyarchuk B *et al*: **Origin and diffusion of mtDNA haplogroup X**. *American journal of human genetics* 2003, **73**(5):1178-1190.

120. Thomas MG, Weale ME, Jones AL, Richards M, Smith A, Redhead N, Torroni A, Scozzari R, Gratrix F, Tarekegn A *et al*: **Founding mothers of Jewish communities: geographically separated Jewish groups were independently founded by very few female ancestors**. *American journal of human genetics* 2002, **70**(6):1411-1420.

121. Vigilant L, Stoneking M, Harpending H, Hawkes K, Wilson AC: **African populations and the evolution of human mitochondrial DNA**. *Science (New York, NY* 1991, **253**(5027):1503-1507.

122. Watson E, Forster P, Richards M, Bandelt HJ: **Mitochondrial footprints of human expansions in Africa**. *American journal of human genetics* 1997, **61**(3):691-704.

123. Brakez Z, Bosch E, Izaabel H, Akhayat O, Comas D, Bertranpetit J, Calafell F: **Human mitochondrial DNA sequence variation in the Moroccan population of the Souss area**. *Annals of human biology* 2001, **28**(3):295-307.

124. Brehm A, Pereira L, Kivisild T, Amorim A: **Mitochondrial portraits of the Madeira and Acores archipelagos witness different genetic pools of its settlers**. *Human genetics* 2003, **114**(1):77-86.

125. Fadhlaoui-Zid K, Plaza S, Calafell F, Ben Amor M, Comas D, Bennamar El gaaied A: **Mitochondrial DNA heterogeneity in Tunisian Berbers**. *Annals of human genetics* 2004, **68**(Pt 3):222-233.

126. Monson KL, Miller KWP, Wilson MR, DiZinno JA, Budowle B: **The mtDNA population database: an integrated software and database resource for forensic Comparison**. *Forensic Sci Comm* 2002, **4**.

127. Rando JC, Cabrera VM, Larruga JM, Hernández M, González AM, Pinto F, Bandelt HJ: **Phylogeographic patterns of mtDNA reflecting the colonization of the Canary Islands**. *Annals of human genetics* 1999, **63**(Pt 5):413-428.

128. Rando JC, Pinto F, Gonzalez AM, Hernandez M, Larruga JM, Cabrera VM, Bandelt HJ: **Mitochondrial DNA analysis of northwest African populations reveals genetic exchanges with European, near-eastern, and sub-Saharan populations**. *Annals of human genetics* 1998, **62**(Pt 6):531-550.

129. Chen YS, Olckers A, Schurr TG, Kogelnik AM, Huoponen K, Wallace DC: **mtDNA variation in the South African Kung and Khwe-and their genetic relationships to other African populations**. *American journal of human genetics* 2000, **66**(4):1362-1383.

130. Soodyall H: **Mitochondrial DNA polymorphisms in Southern African populations**. Johannesbrug: University of the Witwatersrand; 1993.

131. Vigilant L, Pennington R, Harpending H, Kocher TD, Wilson AC: **Mitochondria DNA sequences in single hairs from a southern African population**. *Proc Natl Acad Sci U S A* 1989, **86**:9350-9354.

132. Pereira L, Macaulay V, Torroni A, Scozzari R, Prata MJ, Amorim A: **Prehistoric and historic traces in the mtDNA of Mozambique: insights into the Bantu expansions and the slave trade**. *Annals of human genetics* 2001, **65**(Pt 5):439-458.

133. Salas A, Richards M, De la Fé T, Lareu MV, Sobrino B, Sánchez-Diz P, Macaulay V, Carracedo A: **The making of the African mtDNA landscape**. *American journal of human genetics* 2002, **71**(5):1082-1111.

134. Beleza S, Gusmão L, Amorim A, Carracedo A, Salas A: **The genetic legacy of western Bantu migrations**. *Human genetics* 2005, **117**(4):366-375.

135. Plaza S, Salas A, Calafell F, Corte-Real F, Bertranpetit J, Carracedo A, Comas D: **Insights into the western Bantu dispersal: mtDNA lineage analysis in Angola**. *Human genetics* 2004, **115**(5):439-447.

136. Brehm A, Pereira L, Bandelt HJ, Prata MJ, Amorim A: **Mitochondrial portrait of the Cabo Verde archipelago: the Senegambian outpost of Atlantic slave trade**. *Annals of human genetics* 2002, **66**(Pt 1):49-60.

137. Cerny V, Hajek M, Bromova M, Cmejla R, Diallo I, Brdicka R: **MtDNA of Fulani nomads and their genetic relationships to neighboring sedentary populations**. *Human biology; an international record of research* 2006, **78**(1):9-27.

138. Gonzalez AM, Garcia O, Larruga JM, Cabrera VM: **The mitochondrial lineage U8a reveals a Paleolithic settlement in the Basque country**. *BMC genomics* 2006, **7**:124.

139. Graven L, Passarino G, Semino O, Boursot P, Santachiara-Benerecetti S, Langaney A, Excoffier L: **Evolutionary correlation between control region sequence and restriction polymorphisms in the mitochondrial genome of a large Senegalese Mandenka sample**. *Molecular biology and evolution* 1995, **12**(2):334-345.

140. Jackson BA, Wilson JL, Kirbah S, Sidney SS, Rosenberger J, Bassie L, Alie JA, McLean DC, Garvey WT, Ely B: **Mitochondrial DNA genetic diversity among four ethnic groups in Sierra Leone**. *American journal of physical anthropology* 2005, **128**(1):156-163.

141. Černý V, Salas A, Hájek M, Žaloudková M, Brdička R: **A bidirectional corridor in the Sahel-Sudan belt and the distinctive features of the Chad Basin populations: a history revealed by the mitochondrial DNA genome**. *Annals of human genetics* 2007, **71**(Pt 4):433-452.

142. Coia V, Destro-Bisol G, Verginelli F, Battaggia C, Boschi I, Cruciani F, Spedini G, Comas D, Calafell F: **Brief communication: mtDNA variation in North Cameroon: lack of Asian lineages and implications for back migration from Asia to sub-Saharan Africa**. *American journal of physical anthropology* 2005, **128**(3):678-681.

143. Destro-Bisol G, Coia V, Boschi I, Verginelli F, Caglia A, Pascali V, Spedini G, Calafell F: **The analysis of variation of mtDNA hypervariable region 1 suggests that Eastern and Western Pygmies diverged before the Bantu expansion**. *The American naturalist* 2004, **163**(2):212-226.

144. Mateu E, Comas D, Calafell F, Perez-Lezaun A, Abade A, Bertranpetit J: **A tale of two islands: population history and mitochondrial DNA sequence variation of Bioko and Sao Tome, Gulf of Guinea**. *Annals of human genetics* 1997, **61**(Pt 6):507-518.

145. Pinto F, Gonzalez AM, Hernandez M, Larruga JM, Cabrera VM: **Genetic relationship between the Canary Islanders and their African and Spanish ancestors inferred from mitochondrial DNA sequences**. *Annals of human genetics* 1996, **60**(Pt 4):321-330.

146. Trovoada MJ, Pereira L, Gusmao L, Abade A, Amorim A, Prata MJ: **Pattern of mtDNA variation in three populations from Sao Tome e Principe**. *Annals of human genetics* 2004, **68**(Pt 1):40-54.
