# Supplementary material for: Genetic origin, admixture, and asymmetry in maternal and paternal human lineages in Cuba
Source: BMC Evol Biol. 2008 Jul 21;8:213. doi: 10.1186/1471-2148-8-213 (PMC2492877; doi:10.1186/1471-2148-8-213)
Supplement: Additional file 2 — HVS-I sequences found in Cuba with their haplogroup classification and number of (identical) and matches in the datasets used for comparison [file 1471-2148-8-213-S2.doc]

**Additional file 2**. Mitochondrial DNA sequences found in Cuba with their haplogroup classification and number of (identical) matches in the databases used for comparison. Only positions from 16090 to 16365 were taken into account in the comparison databases. Cuban sequences that differed in positions beyond this range are shown as dashed lines in the database comparisons.

|  |  |  |  | DATABASE AMERICA | | | DATABASE AFRICA | | | | | | |  |
| --- | --- | --- | --- | --- | --- | --- | --- | --- | --- | --- | --- | --- | --- | --- |
| HAPLOTYPE ID | HVS-I (16024-16391) | HAPLOGROUP | N | CENTRAL | NORTH | SOUTH | EAST | NORTH | SOUTH | SOUTHEAST | SOUTHWEST | WEST | CENTRAL | TOTAL |
| African |  |  |  | (N = 83) | (N = 1148) | (N = 143) | (N = 835) | (N = 1312) | (N = 264) | (N = 416) | (N = 157) | (N = 1184) | (N = 1202) |
| 1 | 129 148 168 172 187 188G 189 223 230 311 320 | L0a1 | 2 | 0 | 12 | 2 | 17 | 0 | 0 | 2 | 4 | 9 | 20 | 66 |
| 2 | 129 148 168 172 187 188G 189 223 230 278 293 311 320 | L0a1a | 2 | 1 | 2 | 2 | 5 | 1 | 1 | 22 | 4 | 0 | 11 | 49 |
| 3 | 148 172 187 188G 189 223 230 311 320 | L0a2 | 1 | 1 | 1 | 0 | 7 | 0 | 0 | 52 | 4 | 0 | 3 | 68 |
| 4 | 037 126 172 187 189 223 264 270 278 293 301 311 | L1b1 | 1 | 0 | 0 | 0 | 0 | 0 | 0 | 0 | 0 | 0 | 0 | 0 |
| 5 | 093 126 187 189 223 264 270 278 293 311 | L1b1 | 1 | 0 | 3 | 0 | 1 | 1 | 0 | 0 | 0 | 11 | 8 | 24 |
| 6 | 111 126 187 189 223 239 270 278 293 311 | L1b1 | 1 | 0 | 3 | 0 | 0 | 0 | 0 | 0 | 0 | 3 | 5 | 11 |
| 7 | 126 165T 187 189 223 264 270 278 293 311 | L1b1 | 1 | 0 | 0 | 0 | 0 | 0 | 0 | 0 | 0 | 0 | 0 | 0 |
| 8 | 126 187 189 223 256 264 270 278 293 301 311 | L1b1 | 1 | 0 | 0 | 0 | 0 | 0 | 0 | 0 | 0 | 0 | 0 | 0 |
| 9 | 126 187 189 223 264 270 278 293 311 | L1b1 | 2 | 0 | 41 | 10 | 2 | 15 | 0 | 1 | 0 | 36 | 38 | 143 |
| 10 | 126 187 189 223 264 278 293 311 | L1b1 | 2 | 0 | 0 | 0 | 0 | 0 | 0 | 0 | 0 | 1 | 0 | 1 |
| 11 | 129 187 189 223 278 294 360 | L1c | 1 | 0 | 1 | 0 | 0 | 0 | 0 | 0 | 0 | 0 | 0 | 1 |
| 12 | 086 129 187 189 223 241 278 293 294 311 360 | L1c1 | 1 | 0 | 1 | 0 | 0 | 0 | 0 | 0 | 0 | 0 | 0 | 1 |
| 13 | 093 129 187 189 223 278 293 294 311 360 368 | L1c1 | 1 | 0 | 0 | 0 | 0 | 0 | 0 | 0 | 1 | 0 | 0 | 1 |
| 14 | 129 163 187 189 209 223 278 293 294 311 360 | L1c1 | 1 | 1 | 2 | 0 | 3 | 0 | 0 | 2 | 3 | 0 | 4 | 15 |
| 15 | 129 163 187 189 223 278 293 294 298 311 360 | L1c1 | 1 | 0 | 0 | 0 | 0 | 0 | 0 | 0 | 0 | 0 | 0 | 0 |
| 16 | 129 184 187 189 223 278 294 301 311 360 | L1c1 | 1 | 0 | 0 | 0 | 0 | 0 | 0 | 0 | 0 | 0 | 7 | 7 |
| 17 | 129 187 189 223 278 293 294 311 360 | L1c1 | 1 | 0 | 9 | 0 | 0 | 0 | 0 | 1 | 1 | 1 | 2 | 14 |
| 18 | 129 187 189 223 274 278 293 294 311 360 | L1c1a | 1 | 0 | 1 | 1 | 0 | 0 | 0 | 2 | 0 | 0 | 24 | 28 |
| 19 | 093 129 187 189 223 265C 286G 294 311 360 | L1c2 | 2 | 0 | 0 | 0 | 0 | 0 | 0 | 0 | 0 | 0 | 0 | 0 |
| 20 | 129 187 189 223 265C 278 286 288 294 311 360 | L1c2 | 1 | 0 | 0 | 0 | 0 | 0 | 0 | 0 | 0 | 0 | 0 | 0 |
| 21 | 129 187 189 223 265C 286A 294 311 360 | L1c2 | 1 | 0 | 0 | 0 | 0 | 0 | 0 | 0 | 0 | 0 | 0 | 0 |
| 22 | 093 185 189 192+T 223 278 294 309 360 390 | L2a | 1 | 0 | 0 | 0 | 0 | 0 | 0 | 0 | 0 | 0 | 0 | 0 |
| 23 | 093 189 223 264 278 294 309 390 | L2a | 1 | 0 | 0 | 0 | 0 | 0 | 0 | 0 | 0 | 5 | 0 | 5 |
| 24 | 093 189 223 278 294 390 | L2a | 1 | 0 | 0 | 0 | 2 | 0 | 0 | 0 | 0 | 0 | 0 | 2 |
| 25 | 093 223 256 278 294 309 390 | L2a | 1 | 0 | 1 | 0 | 2 | 0 | 0 | 0 | 1 | 0 | 0 | 4 |
| 26 | 183C 189 223 278 294 309 390 | L2a | 1 | 0 | 2 | 0 | 0 | 0 | 0 | 0 | 0 | 1 | 1 | 4 |
| 27 | 189 193+C 223 278 291 294 309 390 | L2a | 1 | 0 | 0 | 0 | 0 | 0 | 0 | 0 | 0 | 0 | 0 | 0 |
| 28 | 189 223 235 278 294 390 | L2a | 2 | 0 | 0 | 0 | 0 | 0 | 0 | 0 | 0 | 0 | 0 | 0 |
| 29 | 189 223 278 294 297 309 390 | L2a | 1 | 0 | 0 | 0 | 0 | 0 | 0 | 0 | 0 | 0 | 0 | 0 |
| 30 | 189 223 278 294 309 390 | L2a | 1 | 2 | 12 | 1 | 26 | 3 | 0 | 2 | 0 | 13 | 6 | 65 |
| 31 | 223 278 290G 294 355 390 | L2a | 1 | 0 | 0 | 0 | 0 | 0 | 0 | 0 | 0 | 0 | 0 | 0 |
| 32 | 223 278 292 294 311 390 | L2a | 2 | 0 | 1 | 0 | 0 | 0 | 0 | 0 | 0 | 0 | 0 | 1 |
| 33 | 223 278 294 309 368 390 | L2a | 2 | 3 | 48 | 8 | 4 | 12 | 0 | 10 | 1 | 23 | 20 | 129 |
| 34 | 223 278 294 309 390 | L2a | 2 | --- | --- | --- | --- | --- | --- | --- | --- | --- | --- | --- |
| 35 | 223 278 294 309 390 391 | L2a | 1 | --- | --- | --- | --- | --- | --- | --- | --- | --- | --- | --- |
| 36 | 223 278 294 390 | L2a | 1 | 0 | 9 | 1 | 0 | 1 | 0 | 0 | 2 | 3 | 5 | 21 |
| 37 | 193 213 223 239 260 278 294 309 390 | L2a1 | 1 | 0 | 0 | 0 | 0 | 0 | 0 | 0 | 0 | 0 | 0 | 0 |
| 38 | 213 223 278 294 309 390 | L2a1 | 1 | 0 | 0 | 0 | 0 | 0 | 0 | 0 | 0 | 2 | 1 | 3 |
| 39 | 129 223 278 286 294 309 390 | L2a1a | 1 | 0 | 0 | 0 | 0 | 0 | 0 | 0 | 0 | 0 | 0 | 0 |
| 40 | 223 278 286 294 309 390 | L2a1a | 2 | 0 | 14 | 0 | 3 | 2 | 0 | 35 | 3 | 4 | 3 | 64 |
| 41 | 114A 129 213 223 234 278 390 | L2b | 1 | 0 | 0 | 0 | 0 | 0 | 0 | 0 | 0 | 0 | 0 | 0 |
| 42 | 114A 129 213 223 278 355 362 390 | L2b1 | 1 | 0 | 17 | 0 | 0 | 0 | 0 | 0 | 5 | 6 | 3 | 31 |
| 43 | 168 223 278 390 | L2c | 1 | 0 | 0 | 0 | 0 | 0 | 0 | 0 | 0 | 0 | 0 | 0 |
| 44 | 223 264 278 390 | L2c | 1 | 0 | 5 | 0 | 0 | 0 | 0 | 0 | 0 | 5 | 3 | 13 |
| 45 | 223 278 390 | L2c | 1 | 0 | 18 | 4 | 0 | 3 | 1 | 0 | 0 | 59 | 12 | 97 |
| 46 | 093 189 223 264 278 390 | L2c2 | 2 | 0 | 0 | 0 | 0 | 0 | 0 | 0 | 0 | 0 | 0 | 0 |
| 47 | 048 124 163 223 278 362 | L3b | 1 | 1 | 0 | 0 | 0 | 0 | 0 | 0 | 0 | 0 | 0 | 1 |
| 48 | 124 183C 187A 189 223 278 362 | L3b | 2 | 0 | 0 | 0 | 0 | 0 | 0 | 0 | 0 | 0 | 0 | 0 |
| 49 | 124 189 223 278 311 362 | L3b | 1 | 0 | 0 | 0 | 0 | 0 | 0 | 0 | 0 | 0 | 1 | 1 |
| 50 | 124 223 270 278 362 | L3b | 2 | 0 | 0 | 0 | 0 | 0 | 0 | 0 | 1 | 0 | 0 | 1 |
| 51 | 124 223 278 362 | L3b | 5 | 1 | 35 | 1 | 3 | 4 | 0 | 1 | 2 | 59 | 34 | 140 |
| 52 | 145 223 278 362 | L3b1 | 1 | 0 | 5 | 0 | 0 | 0 | 0 | 0 | 0 | 1 | 0 | 6 |
| 53 | 093 124 223 | L3d | 2 | 0 | 1 | 0 | 0 | 0 | 0 | 1 | 0 | 3 | 0 | 5 |
| 54 | 111 124 223 | L3d | 2 | 0 | 6 | 1 | 0 | 1 | 0 | 0 | 0 | 9 | 4 | 21 |
| 55 | 124 148 223 259 293 362 | L3d | 1 | 0 | 0 | 0 | 0 | 0 | 0 | 0 | 0 | 0 | 0 | 0 |
| 56 | 124 166 223 | L3d | 2 | 0 | 2 | 0 | 0 | 1 | 0 | 0 | 0 | 3 | 7 | 13 |
| 57 | 124 223 | L3d | 2 | 2 | 8 | 1 | 2 | 3 | 0 | 3 | 0 | 20 | 12 | 51 |
| 58 | 124 223 319 | L3d1 | 1 | 0 | 3 | 0 | 8 | 0 | 0 | 12 | 1 | 1 | 1 | 26 |
| 59 | 124 223 256 291 368 | L3d2 | 1 | 0 | 0 | 0 | 0 | 0 | 0 | 0 | 0 | 1 | 0 | 1 |
| 60 | 124 223 256 368 | L3d2 | 2 | 0 | 11 | 2 | 4 | 2 | 0 | 0 | 1 | 14 | 4 | 38 |
| 61 | 124 183C 189 223 278 304 311 | L3d3 | 1 | 1 | 2 | 0 | 0 | 0 | 0 | 0 | 3 | 0 | 2 | 8 |
| 62 | 183C 189 223 260 327 | L3e1 | 1 | 0 | 1 | 0 | 0 | 0 | 0 | 0 | 0 | 0 | 0 | 1 |
| 63 | 172 185 223 311 327 | L3e1a | 1 | 0 | 0 | 0 | 0 | 0 | 0 | 0 | 0 | 0 | 0 | 0 |
| 64 | 192 223 320 | L3e2 | 3 | 0 | 0 | 0 | 0 | 0 | 0 | 0 | 0 | 0 | 0 | 0 |
| 65 | 223 271 320 | L3e2 | 1 | 0 | 0 | 0 | 0 | 0 | 0 | 0 | 0 | 0 | 0 | 0 |
| 66 | 223 320 | L3e2 | 5 | 0 | 11 | 0 | 0 | 1 | 1 | 0 | 0 | 13 | 18 | 44 |
| 67 | 172 183C 189 223 320 | L3e2b | 1 | 2 | 22 | 0 | 2 | 0 | 0 | 1 | 4 | 4 | 9 | 44 |
| 68 | 172 189 223 320 | L3e2b | 1 | 0 | 11 | 10 | 1 | 2 | 5 | 2 | 0 | 9 | 7 | 47 |
| 69 | 223 265T | L3e3 | 2 | 0 | 15 | 0 | 5 | 1 | 1 | 10 | 2 | 0 | 7 | 41 |
| 70 | 051 223 264 | L3e4 | 1 | 3 | 5 | 1 | 1 | 1 | 0 | 1 | 0 | 27 | 5 | 44 |
| 71 | 209 223 278 311 | L3f | 1 | 0 | 0 | 0 | 0 | 0 | 0 | 0 | 0 | 0 | 0 | 0 |
| 72 | 209 223 311 | L3f | 2 | 0 | 3 | 1 | 1 | 5 | 0 | 3 | 8 | 2 | 12 | 35 |
| 73 | 209 223 235 292 311 | L3f1 | 1 | 0 | 0 | 0 | 0 | 0 | 0 | 0 | 0 | 2 | 0 | 2 |
| 74 | 209 223 292 311 | L3f1 | 1 | 0 | 7 | 0 | 12 | 3 | 0 | 0 | 0 | 3 | 18 | 43 |
| 75 | 179 183C 189 223 239 311 320 362 | L4* | 1 | 0 | 2 | 0 | 0 | 0 | 0 | 0 | 0 | 0 | 0 | 2 |
| 76 | 051 114 183C 189 192 223 293T 311 316 355 362 | L4g | 1 | 0 | 0 | 0 | 0 | 0 | 0 | 0 | 0 | 0 | 0 | 0 |
| 77 | 172 184 219 234 278 | U6a | 1 | 0 | 0 | 0 | 0 | 0 | 0 | 0 | 0 | 0 | 0 | 0 |
| 78 | 163 164T 172 219 311 | U6b1 | 1 | 0 | 0 | 0 | 0 | 2 | 0 | 0 | 0 | 0 | 0 | 2 |
| 79 | 163 172 219 311 | U6b1 | 3 | 1 | 0 | 0 | 0 | 26 | 0 | 0 | 0 | 0 | 0 | 27 |
|  |  | **TOTAL** | **111** | **19** | **353** | **46** | **111** | **90** | **9** | **163** | **51** | **353** | **317** | **1512** |

|  |  | | | |  |  | DATABASE AMERICA | | |  |
| --- | --- | --- | --- | --- | --- | --- | --- | --- | --- | --- |
| HAPLOTYPE ID | HVS-I (range 16024-16391) | | | | HAPLOGROUP | N | CENTRAL | NORTH | SOUTH | TOTAL |
| **Native American** |  | | | |  |  | (N = 485) | (N = 2005) | (N = 1657) |
| 80 | 223 290 319 362 | | | | A2 | 1 | 3 | 10 | 33 | 46 |
| 81 | 114 223 290 319 362 | | | | A2 | 2 | 0 | 0 | 0 | 0 |
| 82 | 093 223 290 319 362 | | | | A2 | 2 | 0 | 2 | 0 | 2 |
| 83 | 092 223 290 319 362 | | | | A2 | 1 | 0 | 0 | 0 | 0 |
| 84 | 223 240 278 290 319 362 | | | | A2 | 1 | 0 | 0 | 0 | 0 |
| 85 | 111 223 290 319 362 | | | | A2 | 5 | 45 | 161 | 50 | 256 |
| 86 | 111 223 290 319 362 390 | | | | A2 | 6 | --- | --- | --- | 0 |
| 87 | 111 126 223 290 319 362 | | | | A2 | 1 | 3 | 0 | 0 | 3 |
| 88 | 111 126 223 256 290 319 362 | | | | A2 | 4 | 0 | 5 | 0 | 5 |
| 89 | 111 126 223 256 290 319 362 390 | | | | A2 | 1 | --- | --- | --- | 0 |
| 90 | 111 126 223 290 311 319 362 | | | | A2 | 1 | 0 | 0 | 0 | 0 |
| 91 | 111 126 223 290 311 319 362 383 | | | | A2 | 1 | --- | --- | --- | 0 |
| 92 | 111 126 223 256 290 311 319 362 | | | | A2 | 1 | 0 | 0 | 0 | 0 |
| 93 | 111 218 223 290 319 362 | | | | A2 | 1 | 0 | 0 | 0 | 0 |
| 94 | 111 218 223 290 294 319 362 | | | | A2 | 2 | 0 | 0 | 0 | 0 |
| 95 | 111 175 223 290 300 319 362 | | | | A2 | 4 | 1 | 1 | 0 | 2 |
| 96 | 086 111 175 223 259 290 300 311 319 362 | | | | A2 | 1 | 0 | 0 | 0 | 0 |
| 97 | 111 189 223 290 319 336 362 | | | | A2 | 6 | 0 | 0 | 0 | 0 |
| 98 | 111 223 266 290 319 362 | | | | A2 | 6 | 0 | 1 | 4 | 5 |
| 99 | 111 187 223 290 319 362 | | | | A2 | 1 | 25 | 2 | 0 | 27 |
| 100 | 083 092 111 223 256 274 290 319 362 | | | | A2 | 1 | 0 | 0 | 0 | 0 |
| 101 | 111 213 223 290 319 362 | | | | A2 | 2 | 0 | 0 | 3 | 3 |
| 102 | 111 223 287G 290 319 362 | | | | A2 | 1 | 0 | 0 | 0 | 0 |
| 103 | 098 106 111 223 290 319 | | | | A2 | 1 | 0 | 0 | 0 | 0 |
| 104 | 084 111 181 215 223 249 290 319 362 | | | | A2 | 1 | 0 | 0 | 0 | 0 |
| 105 | 111 223 290 319 344 362 | | | | A2 | 1 | 0 | 0 | 0 | 0 |
| 106 | 092 182C 183C 189 217 249 312 344 | | | | B2 | 1 | 0 | 1 | 0 | 1 |
| 107 | 156 166 183C 189 217 | | | | B2 | 1 | 0 | 0 | 0 | 0 |
| 108 | 183C 189 194C 195 258C 217 | | | | B2 | 1 | 0 | 0 | 0 | 0 |
| 109 | 182C 183C 189 217 257 | | | | B2 | 2 | 0 | 0 | 0 | 0 |
| 110 | 223 298 325 327 | | | | C1 | 7 | 19 | 111 | 195 | 325 |
| 111 | 069 223 298 325 327 | | | | C1 | 1 | --- | --- | --- | 0 |
| 112 | 093 223 298 325 327 | | | | C1 | 1 | 1 | 0 | 5 | 6 |
| 113 | 223 274 298 325 327 | | | | C1 | 1 | 0 | 5 | 0 | 5 |
| 114 | 129 223 298 325 327 | | | | C1 | 1 | 0 | 2 | 4 | 6 |
| 115 | 051 223 298 319 325 327 335 | | | | C1d | 1 | 0 | 0 | 0 | 0 |
| 116 | 051 086 124 142 223 258C 265C 292 298 325 327 | | | | C1d | 1 | 0 | 0 | 0 | 0 |
| 117 | 192 223 325 362 | | | | D1 | 4 | 0 | 0 | 0 | 0 |
| 118 | 223 325 362 | | | | D1 | 4 | 5 | 34 | 35 | 74 |
|  |  |  |  | TOTAL | | 81 | 102 | 766 | 329 | 766 |

|  |  |  |  | DATABASE EUROPE | | | | |  | ATLANTIC ISLANDS |  |
| --- | --- | --- | --- | --- | --- | --- | --- | --- | --- | --- | --- |
| HAPLOTYPE ID | HVS-I (range 16024-16391) | HAPLOGROUP | N | SOUTHWEST | WEST | CENTRAL | SOUTHEAST | NORTH | TOTAL | (Madeira/Açores/Canary) | TOTAL |
| European |  |  |  | 3154 | 959 | 2123 | 1356 | 1053 | 8645 | 778 | 9423 |
| 119 | 218 311 | H | 2 | 0 | 0 | 0 | 0 | 0 | 0 | 0 | 0 |
| 120 | 148 170C | H | 1 | 0 | 0 | 0 | 0 | 0 | 0 | 0 | 0 |
| 121 | 176 239 | H | 1 | 0 | 0 | 0 | 0 | 0 | 0 | 0 | 0 |
| 122 | 262 | H | 1 | 1 | 0 | 1 | 0 | 0 | 2 | 0 | 2 |
| 123 | 311 | H | 5 | 43 | 13 | 48 | 45 | 14 | 163 | 6 | 169 |
| 124 | CRS | H | 5 | 542 | 156 | 300 | 149 | 134 | 1281 | 142 | 1423 |
| 125 | 209 304 | H | 2 | 1 | 0 | 0 | 0 | 0 | 1 | 0 | 1 |
| 126 | 304 | H | 2 | 43 | 21 | 41 | 51 | 12 | 168 | 2 | 170 |
| 127 | 162 | H | 1 | 6 | 4 | 14 | 4 | 13 | 41 | 2 | 43 |
| 128 | 176 | H | 1 | 13 | 2 | 0 | 0 | 0 | 15 | 3 | 18 |
| 129 | 266 | H | 1 | 3 | 0 | 1 | 0 | 1 | 5 | 0 | 5 |
| 130 | 129 172 223 311 | I1 | 1 | 7 | 4 | 6 | 3 | 11 | 31 | 1 | 32 |
| 131 | 069 126 | J* | 1 | 48 | 46 | 68 | 40 | 42 | 244 | 13 | 257 |
| 132 | 069 126 291 | J* | 2 | 4 | 0 | 1 | 0 | 0 | 5 | 0 | 5 |
| 133 | 069 126 311 | J* | 1 | 5 | 0 | 1 | 0 | 0 | 6 | 4 | 10 |
| 134 | 069 126 318C | J* | 1 | 0 | 0 | 0 | 0 | 0 | 0 | 0 | 0 |
| 135 | 069 126 278 366 | J* | 1 | 5 | 0 | 0 | 0 | 0 | 5 | 2 | 7 |
| 136 | 069 126 145 231 261 | J2a | 1 | 3 | 5 | 20 | 1 | 5 | 34 | 8 | 42 |
| 137 | 069 126 145 222 256 261 278 | J1b | 1 | 2 | 0 | 0 | 0 | 0 | 2 | 0 | 2 |
| 138 | 069 126 193 278 | J2 | 1 | 14 | 2 | 6 | 1 | 1 | 24 | 4 | 28 |
| 139 | 224 270 311 | K | 1 | 17 | 0 | 0 | 0 | 0 | 17 | 0 | 17 |
| 140 | 222 224 265 270 311 | K | 1 | 0 | 0 | 0 | 0 | 0 | 0 | 0 | 0 |
| 141 | 126 192 294 296 | T* | 1 | 0 | 0 | 0 | 0 | 0 | 0 | 0 | 0 |
| 142 | 126 163 186 189 261 294 | T1a | 2 | 3 | 0 | 0 | 0 | 0 | 3 | 1 | 4 |
| 143 | 126 294 296 304 | T2 | 2 | 34 | 15 | 44 | 11 | 17 | 121 | 19 | 140 |
| 144 | 126 292 294 | T | 2 | 16 | 1 | 1 | 2 | 0 | 20 | 4 | 24 |
| 145 | 129 169 172 189 | U* | 1 | 0 | 0 | 0 | 0 | 0 | 0 | 3 | 3 |
| 146 | 274 | U* | 1 | 0 | 0 | 1 | 0 | 0 | 1 | 0 | 1 |
| 147 | 179 356 | U4 | 1 | 7 | 3 | 18 | 7 | 0 | 35 | 0 | 35 |
| 148 | 092 179 192 356 | U4 | 1 | 0 | 0 | 0 | 0 | 0 | 0 | 0 | 0 |
| 149 | 134 266 356 | U4a2 | 1 | 0 | 0 | 0 | 0 | 0 | 0 | 0 | 0 |
| 150 | 086 134 356 | U4a2 | 2 | 0 | 0 | 0 | 0 | 0 | 0 | 0 | 0 |
| 151 | 192 239 256 270 | U5a | 2 | 0 | 2 | 0 | 0 | 0 | 2 | 0 | 2 |
| 152 | 291 298 | V | 2 | 2 | 0 | 2 | 1 | 0 | 5 | 3 | 8 |
| 153 | 223 292 | W | 1 | 11 | 7 | 11 | 2 | 6 | 37 | 3 | 40 |
|  |  | TOTAL | 53 | 830 | 281 | 584 | 317 | 256 | 2268 | 220 | 2488 |
