# Supplementary material for: Genetic origin, admixture, and asymmetry in maternal and paternal human lineages in Cuba
Source: BMC Evol Biol. 2008 Jul 21;8:213. doi: 10.1186/1471-2148-8-213 (PMC2492877; doi:10.1186/1471-2148-8-213)
Supplement: Additional file 3 — References for the studies on admixed populations used for comparisons. [file 1471-2148-8-213-S3.doc]

**Additional file 3**. Previous studies on admixed populations used for comparisons.

|  | **Population** | **Type** | **Reference** | **mtDNA** | | | | | **Y-chromosome** | | | |
| --- | --- | --- | --- | --- | --- | --- | --- | --- | --- | --- | --- | --- |
|  |  |  |  | Native American | European | | African | Unknown  /Other | Native American | European | African | Unknown  /Other |
|  | | | | | | | | | | | | |
| **USA** | USA | African American | [1] | 2 | 5.1 | | 91.5 | 1.4 | NA | NA | NA | NA |
|  | | | | | | | | | | | | |
| **Caribbean** | Jamaica | African American | [2] | NA | NA | | NA | NA | 0 | 41.7 | 58.3 | 0 |
|  | Dominan Republic | General urban | [3] | 51.5 | 9.1 | | 39.4 | 0 | NA | NA | NA | NA |
|  | Puerto Rico | General urban | [4] | 61.1 | 11.4 | | 27.5 | 0 | NA | NA | NA | NA |
|  | St. Thomas | African American | [2] | NA | NA | | NA | NA | 0 | 19.4 | 80.6 | 0 |
|  | St. Kitts | African American | [2] | 0 | 0 | | 100 | 0 | 0 | 11.7 | 88.3 | 0 |
|  | Dominica | African American | [2] | 28.0 | 0 | | 72.0 | 0 | 0 | 65.9 | 34.1 | 0 |
|  | St. Lucia | African American | [2] | 0 | 3.6 | | 92.8 | 3.6 | 0 | 54.2 | 45.8 | 0 |
|  | St. Vincent | African American | [2] | 1.8 | 5.5 | | 92.7 | 0 | 0 | 22.5 | 77.5 | 0 |
|  | Grenada | African American | [2] | 0 | 7.8 | | 86.3 | 5.9 | 0 | 18.3 | 81.7 | 0 |
|  | Trinidad | African American | [2] | 3.6 | 7.3 | | 78.2 | 10.9 | 0 | 24.0 | 76.0 | 0 |
|  | | | | | | | | | | | | |
| **Latin** | North-Central Mexico | General urban | [5] | 89.1 | 5.4 | 4.5 | | 0.6 | NA | NA | NA | NA |
| **America** | Garifuna, Belize | African American | [6] | 0 | 0 | 100 | | 0 | NA | NA | NA | NA |
|  | Garifuna, Honduras | African American | [7] | 15.9 | 0 | 84.1 | | 0 | NA | NA | NA | NA |
|  | Chocó, Colombia | African American | [7] | 16.3 | 0 | 83.7 | | 0 | NA | NA | NA | NA |
|  | Medellín, Colombia | General urban | [8] | 1.0 | 94.0 | 5.0 | | 0 | 90.0 | NA | NA | NA |
|  | Brazil | Middle and upper-middle class, mainly whites | [9, 10] | 33.2 | 38.9 | 27.9 | | 0 | 0 | 97.5 | 2.5 | 0 |
|  | Belém, Brazil | General urban | [11] | 57.4 | 40.0 | 2.6 | | 0 | 3.8 | 96.2 | 0 | 0 |
|  | Brazil | Urban whites | [12] | NA | NA | NA | | NA | 0 | 98.0 | 2.0 | 0 |
|  | Salvador, Brazil | African American | [12] | NA | NA | NA | | NA | 4 | 28.0 | 54.0 | 14.0 |
|  | Ribeirao Preto, Brazil | African American | [12] | NA | NA | NA | | NA | 0 | 44.7 | 50.0 | 5.3 |
|  | Barra, Brazil | African American | [12] | NA | NA | NA | | NA | 0 | 22.7 | 77.3 | 0 |
|  | Sao Gonzalo, Brazil | African American | [12] | NA | NA | NA | | NA | 0 | 48.1 | 47 | 5.9 |
|  | Macapá, Brazil | African American | [13] | 46.7 | 0 | 53.3 | | 0 | 6.7 | 56.7 | 36.7 | 0 |
|  | Rio de Janeiro, Brazil | General urban | [14] | NA | NA | NA | | NA | 1.6 | 88.1 | 8.7 | 1.6 |
|  | Santiago, Chile | Low status | [15] | NA | NA | NA | | NA | 15.8 | 84.2 | NA | NA |
|  | Santiago, Chile | High status | [15] | NA | NA | NA | | NA | 6.9 | 93.1 | NA | NA |
|  | La Plata, Argentina | General urban | [16] | NA | NA | NA | | NA | 14.9 | 30.8 | 43.6 | 10.6 |
|  | Tacaurembó, Uruguay | Low status | [17] | 62.5 | 20.8 | 16.7 | | 0 | NA | NA | NA | NA |
|  | Melo, Uruguay | African American | [18] | 28.7 | 19.0 | 52.3 | | 0 | 64.1 | 5.1 | 30.2 | 0.6 |

**References**

1. Allard MW, Polanskey D, Miller K, Wilson MR, Monson KL, Budowle B: **Characterization of human control region sequences of the African American SWGDAM forensic mtDNA data set**. *Forensic science international* 2005, **148**(2-3):169-179.

2. Benn Torres J, Kittles RA, Stone AC: **Mitochondrial and Y chromosome diversity in the English-speaking Caribbean**. *Annals of human genetics* 2007, **71**(Pt 6):782-790.

3. Tajima A, Hamaguchi K, Terao H, Oribe A, Perrotta VM, Baez CA, Arias JR, Yoshimatsu H, Sakata T, Horai S: **Genetic background of people in the Dominican Republic with or without obese type 2 diabetes revealed by mitochondrial DNA polymorphism**. *Journal of human genetics* 2004, **49**(9):495-499.

4. Martinez-Cruzado JC, Toro-Labrador G, Viera-Vera J, Rivera-Vega MY, Startek J, Latorre-Esteves M, Roman-Colon A, Rivera-Torres R, Navarro-Millan IY, Gomez-Sanchez E *et al*: **Reconstructing the population history of Puerto Rico by means of mtDNA phylogeographic analysis**. *American journal of physical anthropology* 2005, **128**(1):131-155.

5. Green LD, Derr JN, Knight A: **mtDNA affinities of the peoples of North-Central Mexico**. *American journal of human genetics* 2000, **66**(3):989-998.

6. Monsalve MV, Hagelberg E: **Mitochondrial DNA polymorphisms in Carib people of Belize**. *Proceedings* 1997, **264**(1385):1217-1224.

7. Salas A, Richards M, Lareu MV, Sobrino B, Silva S, Matamoros M, Macaulay V, Carracedo A: **Shipwrecks and founder effects: divergent demographic histories reflected in Caribbean mtDNA**. *American journal of physical anthropology* 2005, **128**(4):855-860.

8. Carvajal-Carmona LG, Soto ID, Pineda N, Ortiz-Barrientos D, Duque C, Ospina-Duque J, McCarthy M, Montoya P, Alvarez VM, Bedoya G *et al*: **Strong Amerind/white sex bias and a possible Sephardic contribution among the founders of a population in northwest Colombia**. *American journal of human genetics* 2000, **67**(5):1287-1295.

9. Alves-Silva J, da Silva Santos M, Guimaraes PE, Ferreira AC, Bandelt HJ, Pena SD, Prado VF: **The ancestry of Brazilian mtDNA lineages**. *American journal of human genetics* 2000, **67**(2):444-461.

10. Carvalho-Silva DR, Santos FR, Rocha J, Pena SD: **The phylogeography of Brazilian Y-chromosome lineages**. *American journal of human genetics* 2001, **68**(1):281-286.

11. Batista dos Santos SE, Rodrigues JD, Ribeiro-dos-Santos AK, Zago MA: **Differential contribution of indigenous men and women to the formation of an urban population in the Amazon region as revealed by mtDNA and Y-DNA**. *American journal of physical anthropology* 1999, **109**(2):175-180.

12. Abe-Sandes K, Silva WA, Jr., Zago MA: **Heterogeneity of the Y chromosome in Afro-Brazilian populations**. *Human biology; an international record of research* 2004, **76**(1):77-86.

13. Ribeiro-dos-Santos AK, Pereira JM, Lobato MR, Carvalho BM, Guerreiro JF, Batista Dos Santos SE: **Dissimilarities in the process of formation of Curiau, a semi-isolated Afro-Brazilian population of the Amazon region**. *Am J Hum Biol* 2002, **14**(4):440-447.

14. Silva DA, Carvalho E, Costa G, Tavares L, Amorim A, Gusmao L: **Y-chromosome genetic variation in Rio de Janeiro population**. *Am J Hum Biol* 2006, **18**(6):829-837.

15. Cifuentes L, Morales R, Sepulveda D, Jorquera H, Acuna M: **DYS19 and DYS199 loci in a Chilean population of mixed ancestry**. *American journal of physical anthropology* 2004, **125**(1):85-89.

16. Martinez Marignac VL, Bertoni B, Parra EJ, Bianchi NO: **Characterization of admixture in an urban sample from Buenos Aires, Argentina, using uniparentally and biparentally inherited genetic markers**. *Human biology; an international record of research* 2004, **76**(4):543-557.

17. Bonilla C, Bertoni B, Gonzalez S, Cardoso H, Brum-Zorrilla N, Sans M: **Substantial Native American female contribution to the population of Tacuarembo, Uruguay, reveals past episodes of sex-biased gene flow**. *Am J Hum Biol* 2004, **16**(3):289-297.

18. Sans M, Weimer TA, Franco MH, Salzano FM, Bentancor N, Alvarez I, Bianchi NO, Chakraborty R: **Unequal contributions of male and female gene pools from parental populations in the African descendants of the city of Melo, Uruguay**. *American journal of physical anthropology* 2002, **118**(1):33-44.
